# Shared active site architecture between archaeal PolD and multi-subunit RNA polymerases revealed by X-ray crystallography

**DOI:** 10.1038/ncomms12227

**Published:** 2016-08-22

**Authors:** Ludovic Sauguet, Pierre Raia, Ghislaine Henneke, Marc Delarue

**Affiliations:** 1 Unit of Structural Dynamics of Macromolecules, Pasteur Institute and CNRS UMR 3528, 75015 Paris, France; 2 Pierre and Marie Curie University, Paris 6, 75006 Paris, France; 3 Ifremer, UMR 6197, Laboratoire de Microbiologie des Environnements Extrêmes, 29280 Plouzané, France; 4 UBO, UMR 6197, Laboratoire de Microbiologie des Environnements Extrêmes, 29280 Plouzané, France; 5 CNRS, UMR 6197, Laboratoire de Microbiologie des Environnements Extrêmes, 29280 Plouzané, France

## Abstract

Archaeal replicative DNA polymerase D (PolD) constitute an atypical class of DNA polymerases made of a proofreading exonuclease subunit (DP1) and a larger polymerase catalytic subunit (DP2), both with unknown structures. We have determined the crystal structures of *Pyrococcus abyssi* DP1 and DP2 at 2.5 and 2.2 Å resolution, respectively, revealing a catalytic core strikingly different from all other known DNA polymerases (DNAPs). Rather, the PolD DP2 catalytic core has the same ‘double-psi β-barrel' architecture seen in the RNA polymerase (RNAP) superfamily, which includes multi-subunit transcriptases of all domains of life, homodimeric RNA-silencing pathway RNAPs and atypical viral RNAPs. This finding bridges together, in non-viral world, DNA transcription and DNA replication within the same protein superfamily. This study documents further the complex evolutionary history of the DNA replication apparatus in different domains of life and proposes a classification of all extant DNAPs.

## Abstract (web summary)

The structures of many DNA polymerases is known, but PolD was a missing piece. Here, the authors report the crystal structure of this protein and use it to connect the DNA replication machinery with the transcription machinery in the same protein family.

## Graphical abstract

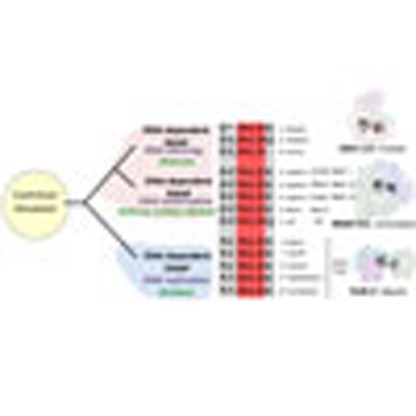

In all forms of cellular life, DNA polymerases (DNAPs) play central roles in genome replication, maintenance and repair, and have therefore been the subject of intensive research for decades^1^. Over the years, all DNAPs have been grouped in different families, using sequence alignments^2^,^3^: PolA, PolB, PolC, PolD, PolX, PolY and reverse transcriptases. Strikingly, nearly all of them belong to one of two different folds^4^, the Klenow-fold (PolA, PolB, PolY and reverse transcriptases) or the Polβ-fold (PolC and PolX). The only class of DNA polymerases left whose structure is unknown and for which the catalytic domain has no assigned fold is PolD.

PolD exists in all Archaea, except Crenarchaea, and is a replicative polymerase responsible for initiating DNA synthesis at both leading and lagging strands^5^,^6^,^7^,^8^. It is composed of a large catalytic subunit (DP2) and a smaller subunit with 3′–5′ proofreading exonuclease activity (DP1)^5^. Apart from the N-terminal regions of its DP1 (ref. 9) (1–50) and DP2 (ref. 10) (50–280) subunits, neither the structure of the catalytic polymerase nor the one of the exonuclease domain has been determined yet. While DP1 is known to belong to the calcineurin-like phosphodiesterase superfamily^11^, DP2 shows no sequence similarity to other proteins with the exception of a short C-terminal zinc-binding motif in eukaryotic Polɛ (ref. 12).

To help resolve the uncertainty concerning the evolutionary origins of D-family DNAP, we determined the crystal structures of two large fragments of both DP1 and DP2 subunits of the *Pyrococcus abyssi* PolD.

## Results

### Crystallization of both DP1 and DP2 catalytic subunits

**Figure 1:**
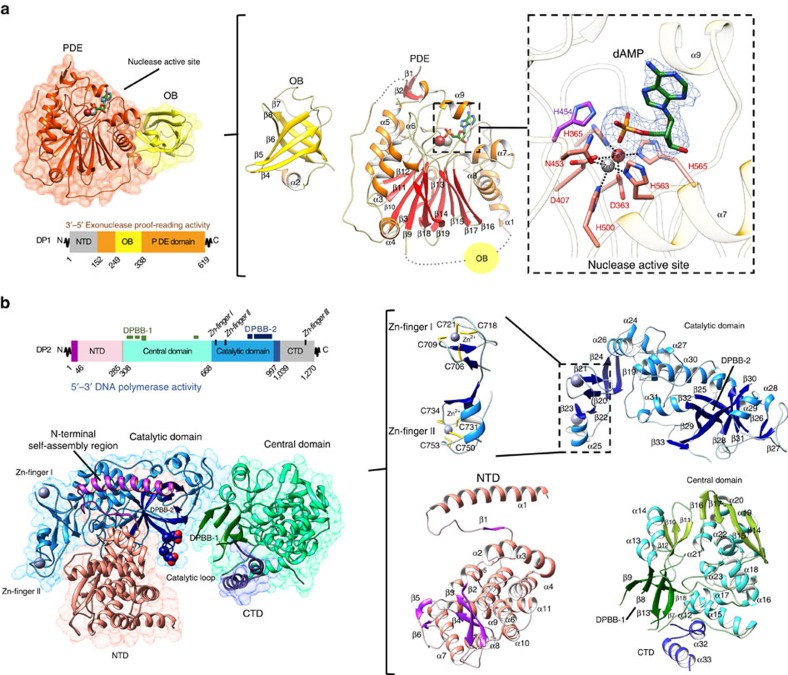
Overview of DP1 and DP2 structures. (**a**) Left: cartoon representation of DP1 (144–622) coloured according to domains: PDE domain, orange; OB domain, yellow. Deleted regions, absent from the construct that was crystallized, are shown in grey. Centre: enlarged views of individual OB and PDE domains coloured by secondary structure. Right: structural details of the DP1 exonuclease active site. Catalytic residues and dAMP are shown as sticks. The blue mesh shows the Fo–Fc omit map electron density surrounding dAMP contoured at 3.0*σ*. (**b**) Left: cartoon representation of DP2 (1–1,050) coloured according to domains: N-terminal self-assembly region, purple; NTD, pink; central domain, cyan; catalytic domain, blue; CTD, dark blue. The aspartic side chains of the conserved D956 and D958 catalytic residues are shown as spheres. Deleted regions, absent from the construct that was crystallized, are shown in grey. Right: enlarged views of individual DP2 domains coloured by secondary structure. Zn^2+^ ions are shown as spheres, and side chains of the cysteine-coordinating residues are shown as sticks.

The structure of DP1 presented here encompasses amino acids 144–622, leaving out a flexible N-terminal region that is not evolutionary conserved and not needed for exonuclease activity (Fig. 1a). The DP2 construct covers amino acids 1–1,050 from DP2 DNAP subunit, leaving out a C-terminal domain (CTD; 1,051–1,270) dedicated to interaction with DP1 (refs 13, 14; Fig. 1b). While the interaction between the two subunits is essential for the full activity of PolD^7^,^14^,^13^, both DP1 and DP2 constructs described here are capable of digesting mispaired 3′–5′ nucleotides and of extending a DNA primer in a templated manner, respectively (Supplementary Fig. 1). Both DP1 and DP2 crystal structures were determined individually by experimental phasing and refined at final resolutions of 2.5 and 2.2 Å, respectively.

### Structure of the PolD DP1 proofreading exonuclease subunit

**Figure 2:**
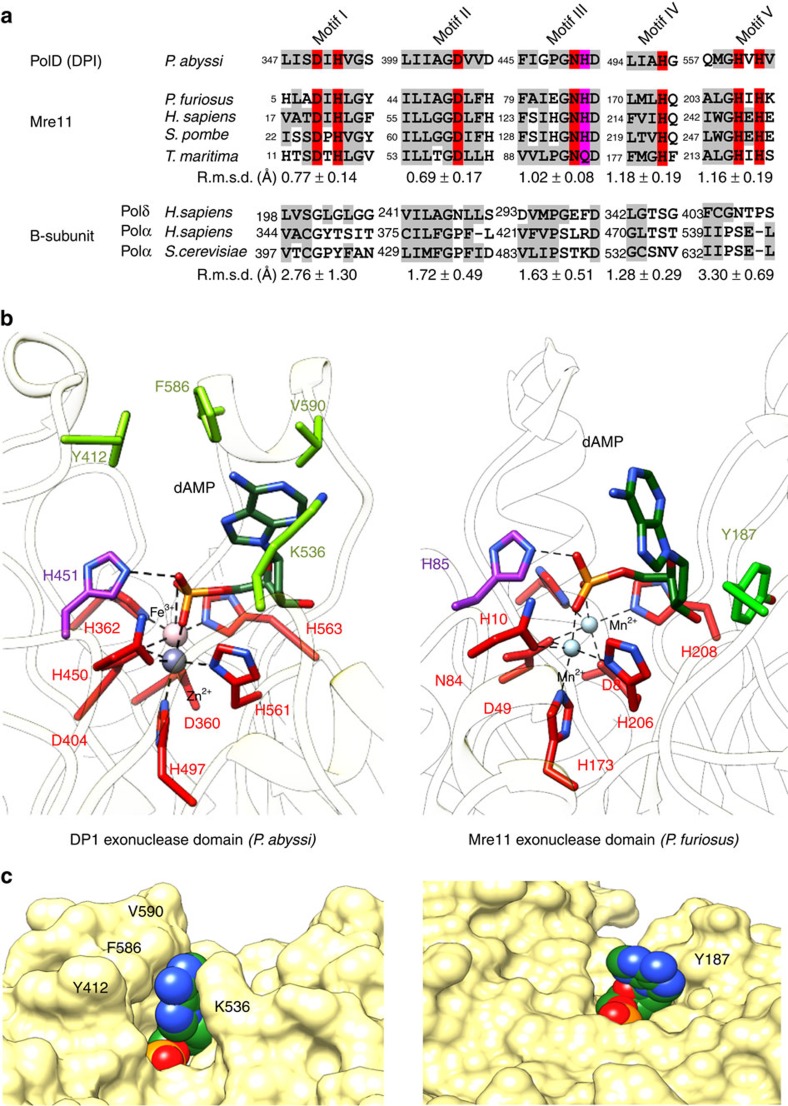
Structure of the DP1 nuclease active site. (**a**) Structure-based alignment of the six most conserved phosphoesterases motifs of the PolD DP1 subunit, with representative structures of Mre11 ((PDBid: 1II7) *P. furiosus*^19^, (PDBid: 3T1L) *Homo sapiens*^67^, (PDBid: 4FBW) *Schizosaccharomyces pombe*^68^ and (PDBid: 3THO) *Thermotoga maritima*^69^), and eukaryotic regulatory B-subunits ((PDBid: 3EOJ) *H. sapiens* Polδ (ref. 26), (PDBid: 4Y97) *H. sapiens* Polα (ref. 27), (PDBid: 3FLO) *S. cerevisiae* Polα (ref. 70). The values indicated below the alignments indicate the r.m.s.d. (in Å) measured on Cα atoms, after superposing each structure on DP1. The r.m.s.d. values are averaged separately for the Mre11 and eukaryotic regulatory B-subunits subgroups of structures. Metal-coordinating residues and the catalytic proton-donor residue are highlighted in red and purple, respectively. (**b**) Comparison of the DP1 and *P. furiosus* Mre11 (PDBid: 1II7 (ref. 19)) nuclease active sites. Metal-coordinating residues (red), proton-donor catalytic residue (purple), dAMP (dark green) and nucleotide base-binding residues (light green) are shown as sticks. (**c**) Compared solvent-accessible surface of the DP1 and *P. furiosus* Mre11 nuclease active sites. The dAMP molecule is in CPK-sphere representation.

DP1 structure shows an insertion of an oligonucleotide/oligosaccharide binding (OB) domain in the N-terminal region of the large calcineurin-like phosphodiesterase (PDE) domain (Fig. 1a). The OB domain (residues 249–338) consists of a five-stranded β-barrel (β_4_–β_8_) wrapped around by helix α_2_ on one side. The PDE domain (residues 152–248 and 339–619) comprises a two-layer β-sheet (β_3_, β_13_–β_17_, and β_9_–β_12_, β_18_–β_19_, respectively) flanked by α-helices α_3_–α_6_ on one side, and by α-helices α_7_–α_8_ on the other side. The PDE domain contains five conserved phosphodiesterase motifs, which form the nuclease active site (Fig. 2a and Supplementary Fig. 2). These motifs are located in loops connecting the core β-strands together and with their flanking α-helices. These loops converge to form a solvent-exposed active site that contains two metal ions, modelled as Fe^3+^ and Zn^2+^ based on anomalous maps using diffraction data measured at the Fe and Zn edges, respectively (Fig. 1a and Supplementary Fig. 3). Metal ions are tetrahedrally coordinated by seven conserved residues distributed in several phosphodiesterase motifs (D363, H365, D407, N453, H500, H563 and H565; Fig. 1a and Supplementary Fig. 4). The oxidation state of the iron ion was modelled as Fe^3+^ based on the fact that the metal-coordination geometry observed in DP1 closely resembles that of di-metal (Fe^3+^/Zn^2+^) phosphatases, including mammalian calcineurin^15^, and the kidney bean purple acid phosphatase^16^. Furthermore, Fe^3+^ has been shown to be required for calcineurin, a structurally similar hydrolase, which undergoes a loss of activity on reduction of Fe^3+^ into Fe^2+^ (ref. 17).

### Structure of the PolD DP1-dAMP complex

Among the calcineurin-like superfamily of proteins, DP1 shows the highest structural similarity with the Mre11 exo-/endonuclease, a key player in DNA repair^18^. To gain insight into the catalytic exonuclease mechanism of PolD, DP1 was co-crystallized together with deoxy-adenosine monophosphate (dAMP) that mimics the 3′-end of the DNA being digested (Figs 1a and 2b). This allows a detailed comparison with the active site of *Pyrococcus furiosus* Mre11 that was also solved in complex with dAMP^19^ (Fig. 2b). *P. abyssi* DP1 and *P. furiosus* Mre11 PDE domains superimpose well (root mean square deviation (r.m.s.d.) value of 2.21 Å calculated over 157 residues; Supplementary Fig. 5) and their metal ions coordinating residues are remarkably conserved (Fig. 2a and Supplementary Fig. 4).

Consistent with what is observed from other ligand-bound PDE structures, dAMP binds mainly via interactions between its phosphate moiety and the coordinated metals, in both *P. furiosus* Mre11 (ref. 19) and DP1 structures (Fig. 2b). The phosphate moiety also interacts with a conserved histidine residue, H85 in Mre11 (ref. 20) and H451 in DP1 (ref. 21), which has been shown to be essential for nuclease activity for re-protonating the 3′-OH of the leaving DNA. These observations suggest that DP1 shares the canonical hydrolase mechanism where the metal ions activate the attacking hydroxide ion, which catalyses hydrolysis of the phosphodiester bond^22^. However, interactions with the adenine base moiety are markedly different between DP1 and *P. furiosus* Mre11. In Mre11, the adenine base of dAMP has little interaction with active site residues, except for a base-stacking interaction with the side chain of Y187 (Fig. 2b)^19^. The Mre11 active site thus exposes the Watson–Crick face of the purine moiety (Fig. 2c), suggesting that the 3′-terminal base is recognized intra-helically^19^, as expected for a nuclease capable of 3′–5′ digestion on paired nucleotides of a 3′-recessed DNA^23^. In contrast to Mre11, the adenine base binds into a narrow groove of the DP1 active site, contributed by the β_8_–α_5_ loop and α-helix α_9_ from one side, and the α_8_–β_13_ loop and α-helix α_7_ from the other side. The adenine base mainly binds through hydrophobic interactions with the side chains of Y415, V593, F589 and the alkyl moiety of K536 (Fig. 2c). As expected for a non-selective nuclease, these residues do not show specific interactions with the adenine base, but instead define a pocket that partially masks the Watson–Crick face of the bound nucleotide (Fig. 2c). This observation provides a structural rationale to biochemical assays showing that DP1 exonuclease prefers mispaired nucleotides over paired nucleotides^24^. The observed preference of PolD DP1 for mispaired nucleotides at the 3′-end is characteristic for a DNAP-associated proofreading exonuclease.

### Evolutionary relationships to eukaryotic DNAPs B-subunits

**Figure 3:**
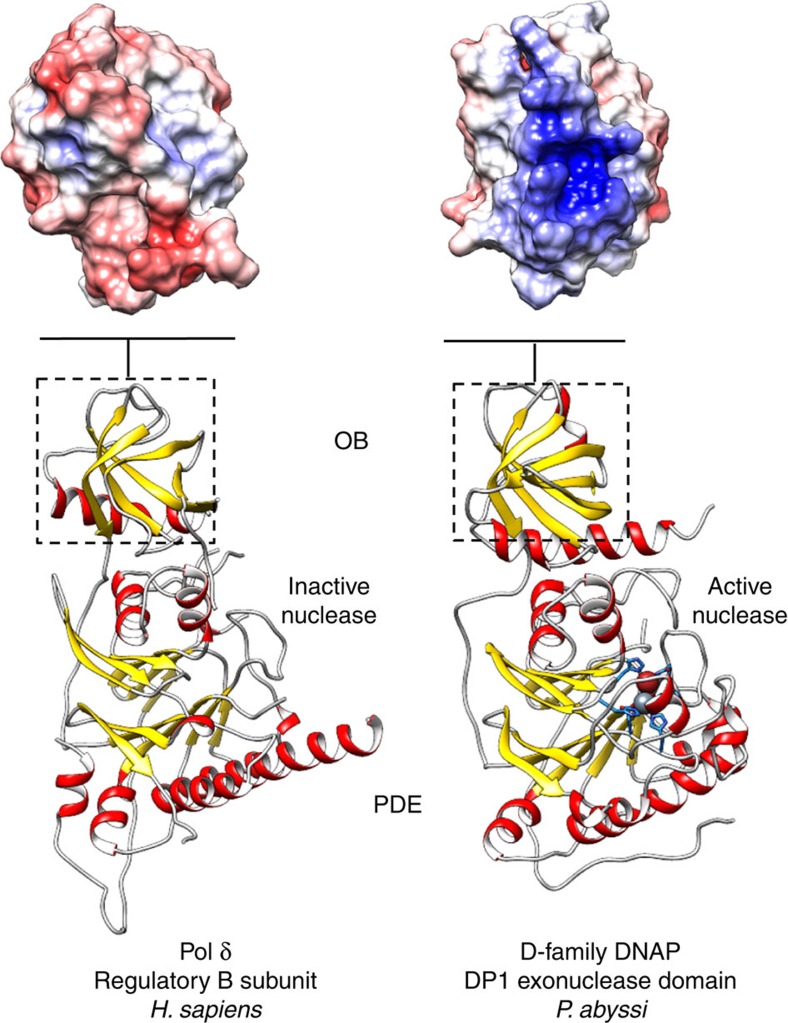
Evolutionary relationships of DP1 to eukaryotic DNAPs B-subunits. Ribbon diagrams highlighting the shared architecture between PolD DP1 and *Homo sapiens* Polδ B-subunit (PDBid: 3EOJ (ref. 26)). Top panels show the electrostatic potentials of DP1 and Polδ OB domains mapped on their solvent-accessible surface at contouring ±5 kT e^−1^. The potential was calculated with APBS^64^.

Consistent with bioinformatics predictions^11^, results of a Dali^25^ search of the protein data bank (Supplementary Fig. 6) show that DP1 shares the strongest overall structural similarity with the regulatory B-subunits of the eukaryotic Polδ (ref. 26) and Polα (ref. 27; Fig. 3). DP1 and the B-subunit of Polδ show a remarkable degree of three-dimensional structural similarity (r.m.s.d. value of 2.56 Å calculated over 322 residues). The region of conservation encompasses both OB (r.m.s.d. value of 1.64 Å calculated over 66 residues) and PDE (r.m.s.d. value of 2.28 Å calculated over 257 residues) domains. However, despite the conserved overall architecture, all DP1 catalytic residues are lost in eukaryotic B-family regulatory subunits (Fig. 2a). In Polδ and Polɛ, the 3′–5′ proofreading activity and DNA polymerizing activities are provided instead by their large A-catalytic subunit, which contains an exonuclease domain. It is noteworthy that the exonuclease domains of Polδ and Polɛ are structurally distinct from that of DP1 and the calcineurin-like PDE superfamily. In addition to their PDE domains, comparison of DP1 and eukaryotic B-subunit OB domains also show substantial structural differences that might account for functional ones. Interestingly, when used in database searches on its own, DP1 OB domain has stronger structural similarity with OB domains of class II-b aminoacyl-tRNA synthetases^28^ and RPA, a DNA single-stranded binding protein involved in replication^29^, rather than with OB domains of eukaryotic B-subunits (Supplementary Fig. 6). The OB-fold architecture displays a conserved binding face well adapted to interact both with RNA and ssDNA^30^, which is also present in DP1 but altered in eukaryotic DNAP B-subunits. Indeed, the surface of DP1 OB domain shows a positive electrostatic potential patch that occupies a location similar to the DNA-binding face observed in other OB domains^30^ (Fig. 3). Polδ and Polα OB domains display no such clusters of conserved positively charged residues suggesting that their oligonucleotides' binding properties are altered.

Alteration of the canonical oligonucleotide-binding surface of their OB domain and inactivation of catalytic nuclease motifs of their PDE domain suggest that eukaryotic DNAP B-subunits have been evolutionary converted to a scaffold mainly responsible for their multi-subunit assembly and perhaps allosteric regulation. Taking into account that B-subunits are indispensable components of all replicative B-family DNAPs and that there is a significant sequence similarity between the C-terminal Zn finger of Polɛ and of PolD DP2, it has been suggested that DP2 is a highly divergent homologue of B-family DNAPs^31^. However, the DP2 structure shares no similarity with known structure of DNAPs, including family-B DNAPs, thereby formally invalidating this hypothesis, as shown below.

### Structure of DP2 DNAP catalytic subunit

The DP2 structure reveals an extended molecule with overall dimensions of 55 × 60 × 110 Å^3^ and four domains: the N-terminal domain (NTD; residues 1–285), the central domain (residues 308–667), the catalytic domain (residues 668–996) and a fragment of the CTD (residues 997–1,039) (Fig. 1b). The most prominent feature of the DP2 architecture is the extensive interaction between the NTD and catalytic domain, which results in a wide interfacial crevice that hosts the DNAP active site, formed by two double-psi β-barrels (DPBBs). Unexpectedly, PolD DP2 and ‘two-barrel' RNA polymerases (RNAPs) share a conserved architecture in their active sites.

**Figure 4:**
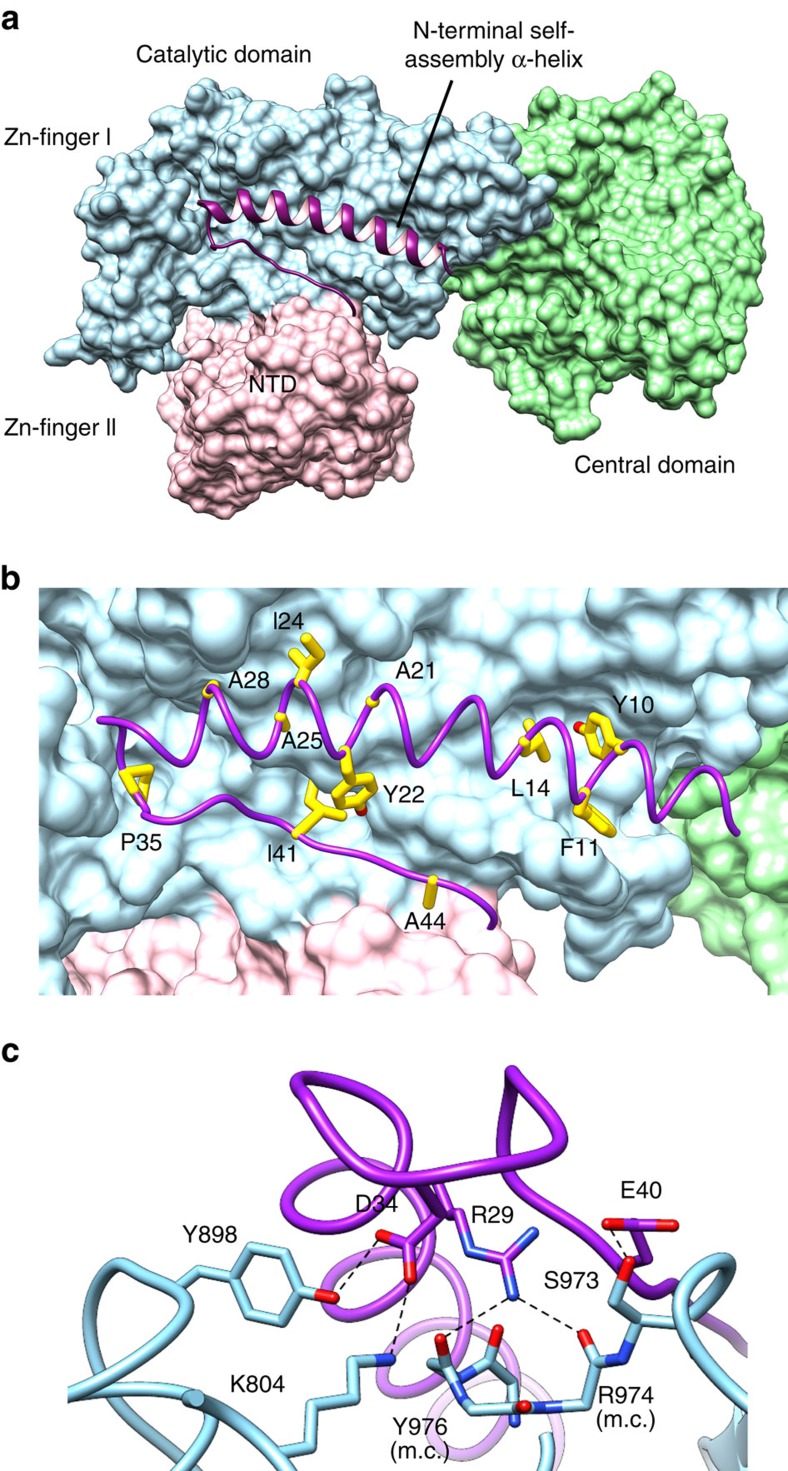
Interactions within the DP2 N-terminal self-assembly region and the catalytic domain. (**a**) Overall view highlighting the involvement of the N-terminal self-assembly α-helix with the catalytic domain. The N-terminal self-assembly α-helix is depicted as purple ribbon, the NTD and catalytic domains as a molecular surface in pink and blue, respectively. (**b**) Hydrophobic interactions at the N-terminal α-helix/catalytic domain interface. Important hydrophobic side chains are shown as yellow sticks. (**c**) Close-up view of the hydrophilic interactions within the N-terminal self-assembly α-helix and the catalytic domain. Side chains are drawn as sticks and hydrogen bonds as dashed lines.

The NTD structure shows a 45-residue long N-terminal extension dedicated to interaction with the catalytic domain (Fig. 1b). This N-terminal extension, named here ‘N-terminal self-assembly region', consists of a long α-helix (α_1_) and a short β-strand (β_1_) that brings together the NTD and catalytic domains through a large interface of 1,700 Å^2^ buried surface area. The majority of the interactions with the catalytic domain are mediated by helix α_1_ and the α_1_–β_1_ connecting loop, which fit within a long and shallow groove formed at the surface of the catalytic domain (Fig. 4a). The chemical nature of this interaction is diverse (Fig. 4b,c) and includes polar contacts, extensive van der Waals contacts between hydrophobic residues, as well as secondary structure interactions between two β-strands, β_1_ (NTD) and β_33_ (catalytic domain). DP2 structure allows a rationalization of previous biochemical studies indicating an intrasubunit interaction between the N terminus and C terminus of the *P. horikoshii* DP2 subunit^10^,^14^. The rest of the NTD structure shows a compact α/β structure that consists of a twisted β-sheet (β_2_–β_4_) and 10 α-helices (α_2_–α_11_). The *P. abyssi* NTD shares a strong similarity with the *P. horikoshii* DP2 (50–290) structure (r.m.s.d. 0.463 Å calculated over 241 Cα) that was exploited at the initial phase of model building (see Methods). Interestingly, the N-terminal self-assembly region was not ordered in the *P. horikoshii* isolated PolD NTD structure, consistent with its dedication to interaction with the catalytic domain.

The catalytic domain is composed of a central six-stranded DPBB (β_25_ and β_28–32_) (Fig. 1b). The first (β_25_) and second (β_28_) β-strands of the DPBB are connected by a 40-residue-long insertion, which forms a compact structure composed of two short β-strands (β_26–27_) and two short α-helices (α_28–29_) that interact with the central domain. The peptide chain connecting the fifth (β_31_) and the sixth (β_32_) β-strands of the barrel forms a right-handed α-helix followed by a 10-residue-long loop, which constitutes the most conserved sequence motif of DP2 (Supplementary Fig. 7). This motif comprises two invariant aspartic residues (D961 and D963), which were previously shown to be catalytically critical for PolD DNAP activity^32^. The DPBB is preceded by a helical region composed of three α-helices (α_24_, α_26–27_) and a tandem repeat of two zinc modules, named Zn-I and Zn-II. The coordinating metals were confidently assigned to zinc by observing strong peaks in the anomalous maps using diffraction data collected at the Zn K-edge, that disappear below this edge (Supplementary Fig. 8). In both cases, Zn^2+^ are tetrahedrally coordinated by two pairs of cysteines located in turns, but apart from that the two zinc-binding modules bear no structural relationship to each other. In particular, zinc module Zn-II has a short helix insertion, an uncommon structural feature shared only with the zinc-binding module of the large subunit of the human transcription factor II-E^33^.

While the N-terminal and catalytic domains interact extensively, the rest of the DP2 structure folds into a central domain that shares a relatively modest 460 Å^2^ interaction surface region with the other domains (Fig. 1b). This results in an increased flexibility of the interfacial region between the central domain and the rest of the protein. As a consequence of this flexibility, several fragments of the peptide chains, all located within this interfacial region, were not modelled due to a lack of interpretable electron density. In particular, the peptide chains connecting the NTD to the central domain (residues 286–307), strands β_7_ to β_8_ (residues 358–364), strands β_8_ to β_9_ (residues 376–392) and strand β_18_ to the catalytic domain (residues 654–668) were not visible in the electron density. This complicated the assignment of a second DPBB subdomain, which faces the first one located in the catalytic domain. Indeed, only five out of six strands (β_7–9_, β_13_ and β_18_) composing this second DPBB have been modelled. To conform to the canonical topology of DPBB, the missing strand should be located in a stretch of 17 missing residues (376–392), which connect the β_8_ and β_9_ strands of the DPBB. Consistently, secondary structure predictions^34^ suggest that this peptide indeed contains a β-strand (Supplementary Fig. 7). The two DPPB subdomains located in the central domain, and the catalytic domain of the DP2 structure are, respectively, named DPBB-1 and DPBB-2, according to their order of occurrence in the primary structure. The location of the two DPBB, with respect to each other, is very similar to that observed in ‘two-barrel' RNAPs (see below). Owing to the presence of several insertions DPBB-1 is spread throughout the central domain that also contains a seven-stranded antiparallel β-sheet (β_10–12_ and β_14–17_) wrapped around by two helices (α_13–14_) from one side and by four helices (α_19–22_) from the other side. This β-sheet is itself interrupted by a small α-helical region formed by four helices (α_15–18_).

The CTD of DP2 (1,000–1,270) is known to be dedicated to an interaction with the DP1 subunit^13^,^14^. For solubility and crystallization purposes, the CTD was truncated by 220 residues (1,051–1,270). The 50 residues of the CTD present in our construct (1,000–1,050) are partly disordered, except for two helices α_32_ and α_33_ (residues 1,011–1,039), which bind next to the interface between the central and catalytic domains through interactions with the DPPB-1 and helix α_32_. This partial structure of the CTD suggests however that the 3′–5′ proofreading DP1 subunit might be localized next to the polymerase active site.

### Evolutionary relationship between PolD and two-barrel RNAPs

When DP2 structure was compared with structures in Protein Data Bank, no significant structural similarity with known DNAP was detected. Instead, PolD shares an unexpected structural homology with the ‘two-barrel' family of RNAP^35^ (Supplementary Fig. 9), which includes multi-subunit transcriptases from all domains of life, homodimeric RNA-silencing pathway RNAPs and atypical RNAPs encoded by some viruses, including some bacteriophages^36^. However, only the DPBB-2 domain was detected by Dali^25^ and the other one, DPBB-1, could only be detected manually. Two-barrel RNAPs share a common catalytic centre that is formed between two DPBBs (here after called DPBB-A and DPBB-B), which contribute distinct amino-acid residues to the active site in an asymmetrical fashion^35^,^37^,^38^. DPBB-A contains a DFDGDE signature^37^, whose aspartate carboxylate residues chelate catalytic Mg^2+^ ions, and DPBB-B contributes two lysine residues that are involved in DNA binding.

**Figure 5:**
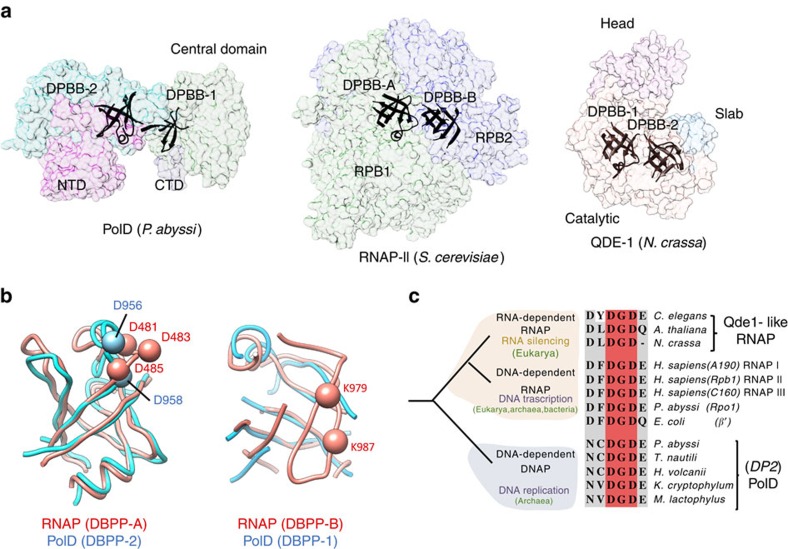
Shared active site architecture between PolD DP2 and ‘two-barrel' RNAPs. (**a**) Overview of the conserved ‘two-barrel' catalytic core in PolD DP2, *S. cerevisiae* RNAP-II (PDBid: 4BBS (ref. 39)) and *Neurospora crassa* QDE-1 (PDBid: 2J7O (ref. 41)). (**b**) Superposition of the DPBB subdomains of PolD (blue) and *S. cerevisiae* RNAP-II (pink). Left: the PolD DPBB-II subdomain is superimposed on the RNAP-II DPBB-A subdomain (Cα r.m.s.d. of 1.72 Å calculated over 73 residues). Cα of the catalytic aspartate residues are shown as spheres. Right: the PolD DPBB-I subdomain is superimposed on the RNAP-II DPBB-B subdomain (Cα r.m.s.d. of 2.21 Å calculated over 42 residues). (**c**) Possible evolutionary relationship between the DNA-dependent DNAP PolD, DNA-dependent RNAPs and RNA-dependent RNAPs. Conserved catalytic motifs are highlighted in a multi-sequence alignment. The alignment was generated using representative protein with a large sequence diversity to illustrate sequence variability (GI accession number): (i) for RNA-dependent RNAPs *Caenorhabditis elegans* (392,886,219), *Arabidopsis thaliana* (42,569,168) and *N. crassa* (85,091,735); (ii) for DNA-dependent RNAPs *Homo sapiens* (4,096,591; 119,610,588; 20,159,751), *Pyrococcus abyssi* (499,169,463) and *Escherichia coli* (983,454,941); and (iii) for D-family DNAPs *P. abyssi* (504,648,395), *Thermococcus nautili* (757,137,858), *Haloferax volcanii* (490,144,762), *Korarchaeum cryptofilum* (501,267,152) and *Methanosarcina mazei* (814,797,709).

Both DPBB subdomains of ‘two-barrel' RNAPs show a remarkable degree of three-dimensional similarity to those of PolD DP2 (Fig. 5a,b). Even in the absence of substrate and Mg^2+^ ions, the catalytic loop in the DPBB-2 of PolD structure can be superposed with the DPBB-A RNAP minimal core structures with a significant overlap (Fig. 3b). In particular, the two mandatory aspartic residues of PolD are conserved and aligned with two out of three canonical catalytic aspartic residues in multi-subunit RNAP (Fig. 5c). Although lacking one β-strand located within a fragment of 17 missing residues, the DPBB-1 domain of PolD structure can also be superposed with the DPBB-B of RNAP. In *Saccharomyces cerevisiae* RNAP-II structure^39^, the corresponding region contains two conserved lysine residues (K979 and K987 in RPB1 subunit), whose side chains point towards the active site and are involved in DNA binding. A multiple-sequence alignment of the corresponding 17 missing residues in PolD structure shows the presence of three basic residues, two lysines (K386 and K392) and one arginine (R389), all located within a highly conserved sequence motif (Supplementary Fig. 7). These conserved positively charged residues might occupy a location similar in PolD to that observed in their RNAP counterparts and therefore contribute to DNA binding.

The finding that PolD and ‘two-barrel' RNAPs share a common catalytic core with similar sequence motifs considerably extends the ‘two-barrel' RNAPs protein family and links it, for the first time, with the DNAPs. Our study also shows that the -DFDGDE- motif is not as strict as previously thought (Fig. 5c) and show that PolD constitute another example of a ‘two-barrel' polymerase that carries the two DPBB domains on the same polypeptide chain, as previously observed on QDE-1, an RNAP that synthesizes siRNA in plants^40^,^41^ (Fig. 5a).

### The ‘two-barrel' architecture defines the DP2 catalytic site

In addition to their structural similarity with the catalytic core of two-barrel RNAPs, converging observations suggest that the DPBB subdomains of PolD host the DNAP active site. Indeed, the peptide chain connecting the fifth (β_31_) and the sixth (β_32_) β-strands of the DPBB-2 comprises two invariant aspartic residues (D956 and D958), which were previously shown to be critical for DNAP catalytical activity, following an extensive alanine-scan site-directed mutagenesis study of all aspartate residues of DP2 (ref. 32). In addition, alteration of the α-helix that precedes these catalytic residues markedly reduced DNA-binding ability and protein stability^42^. Interestingly, in several species including *P. abyssi*, the catalytic loop contains an intein insertion site, which is known to be often located in motifs important for enzymatic activities, especially in archaeal DNA replication proteins^43^.

**Figure 6:**
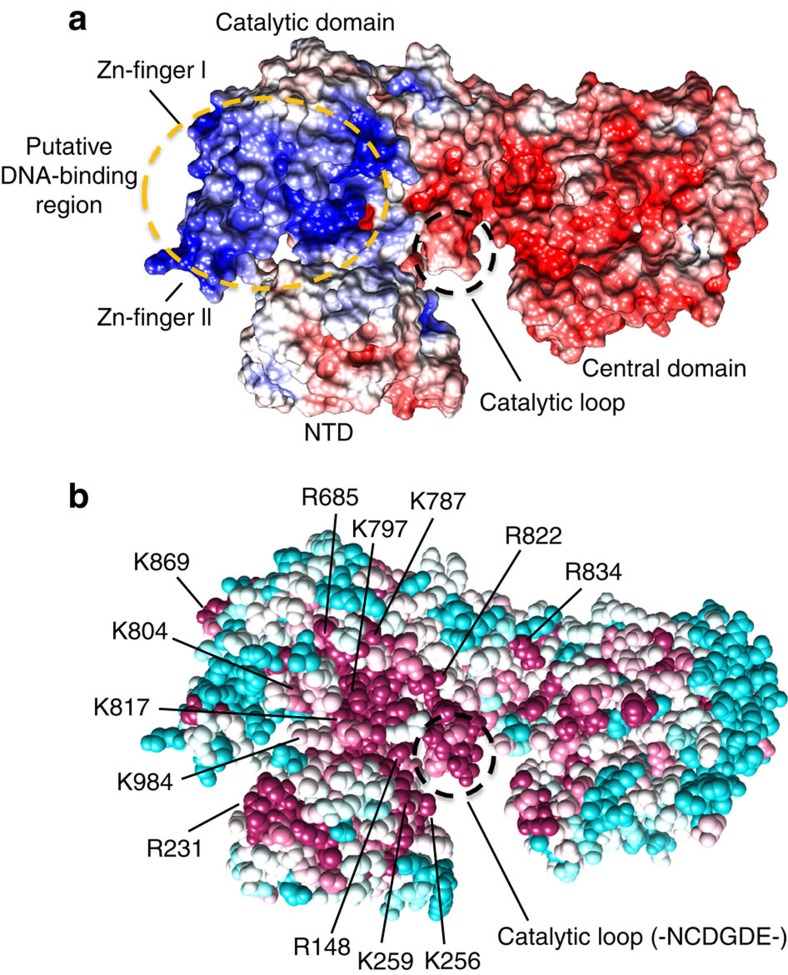
Structural features of PolD DP2 active site. (**a**) Electrostatic potential of DP2, mapped on its solvent-accessible surface at contouring of ±5 kT e^−1^. Positive potential is in blue, negative charge in red. The potential was calculated with APBS^64^. (**b**) Amino-acid conservation mapped on the crystal structure of DP2. The evolutionary conservation analysis of surface residues was performed with the ConSurf^66^ server, based on 12 evolutionary-distant sequences of DP2. Degree of conservation is shown by colour range, from magenta (highest conservation) to cyan (lowest). The structure is shown in space fill representation. Highly conserved residues that might be important for the functional role of DP2 are numbered.

The catalytic loop is solvent-exposed and located within a 55-Å long and 25-Å wide crevice, whose dimensions are ideally suited to orient a 1.5 helical turn long (about 15 bp) duplex B-DNA within the active site. The crevice exposes residues that display a high degree of conservation relative to the rest of the solvent-exposed regions (Fig. 6a). In addition, the electrostatic potential surface of the PolD DP2 subunit presents a highly biased distribution of positively charged residues, located along an arch running from the catalytic loop to the two zinc-binding modules, which could interact with the phosphate backbone of template DNA (Fig. 6b). The crevice is located at the interface between the NTD and catalytic domains, thereby highlighting the functional implications of this inter-domain interaction for DNAP function. Consistently, former biochemical assays revealed that disrupting the N-terminal self-assembly α-helix of *P. abyssi* PolD reduces DNAP processivity^44^.

The DNAP active site shows an increased flexibility compared with the rest of the protein. Some of the conserved motifs in the DPBB-1 subdomain are not seen in the electron density map of the DP2 structure. Comparison with ‘two-barrel' RNAPs suggests that these disordered conserved motifs in DP2 structure also contain important catalytic residues, including basic residues that may be involved in DNA binding (see above). The increased flexibility of catalytic residues might be reflected by the fact that the DP2 structure shows no Mg^2+^ bound in the active site, while structures of ‘two-barrel' RNAPs usually do. The flexibility observed in the active site of DP2 structure might be due to the absence of the DNA substrate, or to the CTD truncation of the DP2 (1–1,050) construct used in this study. Alternatively, the region of the active site might be stabilized on interaction with the DP1 subunit, as suggested by biochemical assays showing that DNAP activity is stimulated on association of the two PolD subunits^7^,^13^,^14^.

Additional studies, including solving a substrate-bound structure of PolD, are required to establish the molecular details of DNA binding, nucleotide selectivity, polymerization and decipher the specificities of D-family DNAPs compared with other DNAP families.

## Discussion

**Figure 7:**
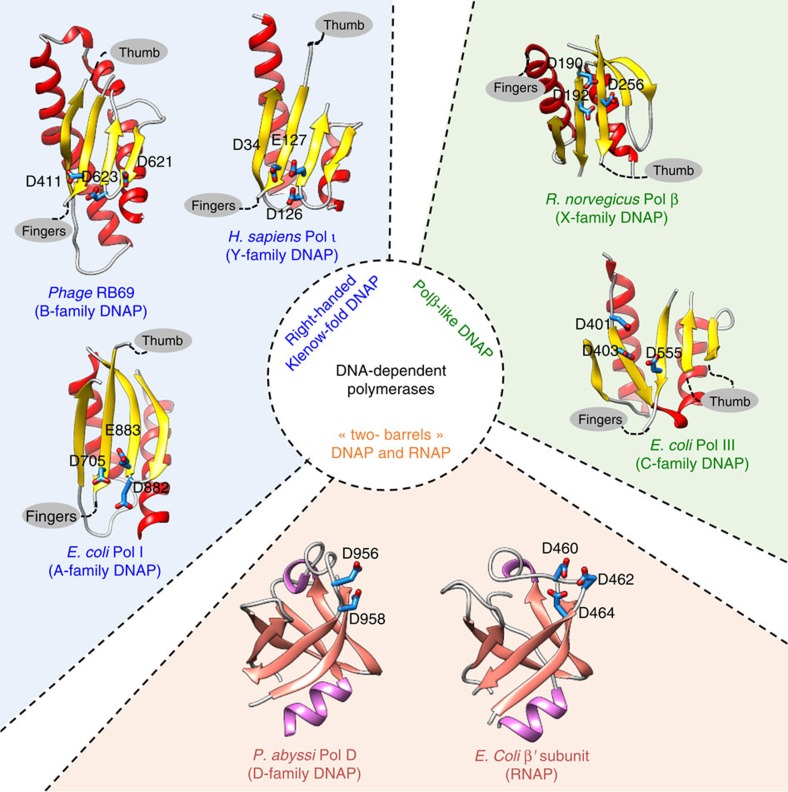
An updated structural classification of all DNA-dependent polymerases. The following crystal structures were used: Polβ from *Rattus norvegicus* (PDBid: 1BPB), Pol III from *Escherichia coli* (PDBid: 2HNH), Polι from *Homo sapiens* (PDBid: 1T3N), PolB from *Enterobacteria phage RB69* (PDBid: 1IH7), Pol I from *E. coli* (PDBid: 1KLN), RNAP β′ subunit from *E. coli* (PDBid: 4MEX) and PolD from *P. abyssi* (this study).

DNAPs have been the subject of extensive structural biology research for decades, which resulted in high-resolution structures of representative DNAPs belonging to different families, following the pioneer work on Klenow fragment of *Escherichia coli* DNA PolI (ref. 45). Up to now, DNAPs of known structures could be divided into two groups based on the structural fold of their catalytic sites^24^. The first group assembles DNAPs that structurally resemble the *E. coli* PolI Klenow-fold^46^, often referred as right-handed polymerases^2^. Their overall fold is characterized by thumb, fingers and palm subdomain, and is shared by A-/B-/Y-family DNAPs, reverse transcriptases and telomerases^47^. The second group, often referred to as Polβ-like polymerases, includes X- and C-families DNAPs, and shares a similar three-dimensional arrangement of catalytic aspartates in the active site^48^,^49^ and a two-metal-ion mechanism^50^ with the first group but has a completely different topology in the palm subdomain (Fig. 7). In many aspects, crystal structures of both DP1 and DP2 subunits revealing that PolD is an atypical DNAP, change this view by linking together DNAP and RNAP. A third group of nucleotide polymerases is created, whose members can perform DNA-dependent RNA or DNA synthesis, and RNA-dependent RNA synthesis.

First, DP1 subunit contains a calcineurin-like phosphodiesterase fold that is responsible for the 3′–5′ proofreading exonuclease activity. In one way, this clearly links PolD to the DNAP world because this fold is present—but catalytically inactive—in the regulatory B-subunit of eukaryotic DNAP. However, in most other structurally characterized DNAPs the catalytically active proofreading domains fold into an α/β structure with a twisted five-stranded mixed β-sheet^51^,^52^,^53^, which shares no structural homology with DP1. While calcineurin-like phosphoesterases includes a diverse range of phosphoesterases^54^, including protein phosphoserine phosphatases, nucleases, nucleotidases, sphingomyelin phosphodiesterases and 2′–3′ cAMP phosphodiesterases, their dedication to DNA proofreading is unique to PolD. Among the calcineurin-like superfamily of proteins, DP1 shows the highest structural similarity with the DNA repair exo-/endonuclease Mre11. Catalytic motifs are remarkably conserved among both enzymes suggesting that they share a common hydrolase catalytic mechanism. However, comparison of DP1 and *P. furiosus* Mre11 dAMP-bound structure^19^ revealed substantial differences in the way the adenine base fits into the active site that may reflect the differences in specificity between both nucleases. In particular, the DP1 active site seems shaped to favour binding of mispaired 3′-terminal nucleotide over paired 3′-terminal nucleotide, a property that is expected for a DNAP-associated proofreading exonuclease. In addition, the dinuclear Fe^3+^/Zn^2+^ metal centre observed in DP1 is another specificity of the PolD structure. Indeed, while asymmetric dinuclear Fe^3+^/Zn^2+^ catalytic centres are common among calcineurin-like phosphoesterases, they are uncommon among nucleases, which prefer symmetrical dinuclear metal centres (Mg^2+^, Mn^2+^ and Zn^2+^)^55^.

Second, the structure of DP2 catalytic subunit shows that it shares no significant structural similarity with known DNAP. Instead, PolD DP2 shares an unexpected structural homology with the ‘two-barrel' family of RNAP^35^, which includes multi-subunit transcriptases from all domains of life, homodimeric RNA-silencing pathway RNAPs and atypical RNAPs encoded by some viruses and phages^36^. The finding that PolD and ‘two-barrel' RNAPs share a common catalytic core with similar sequence motifs considerably extends the ‘two-barrel' RNAPs protein family and links it with the DNAPs (Fig. 7). Also, it shows that the two DPBB subdomains can be part of the same polypeptide chain, contrary to most RNAPs (with the exception of siRNA synthesizing QDE-1 RNAP). This defines a novel paradigm for the classification and possible evolutionary relationships between different types of both RNA and DNAPs. It bridges together, for the first time in the non-viral world, DNA transcription and DNA replication within the same protein superfamily, suggesting that these distantly related polymerases share a common ancestor, which might have been selected for nucleotide polymerization in early forms of life. The capacity of PolD to use RNA-primed DNA might be a property inherited from its common ancestor to RNAP^7^. However, the exact evolutionary history of ‘two-barrel' polymerases remains at this stage speculative, especially as other ‘two-barrel' RNAPs that are found in some large DNA viruses (baculoviruses and nucleocytoplasmic large DNA viruses, including mimiviruses^35^), are very different from their cellular homologues.

Finally, PolDs encoded from Thermococcales possess high processivity, specificity and thermostability properties that confer them a biotechnological potential^56^, which has remained unexploited up to now due to the lack of detailed structural information. The new structures described here pave the way to the complete description of the molecular mechanisms of DNA binding, nucleotide selection and proofreading of this new family of DNAPs that might lead to variants of PolD with better PCR or sequencing properties.

## Methods

### Cloning and protein purification

Residues 144–622 of *P. abyssi* DP1 and residues 1–1,051 of *P. abyssi* DP2 were cloned into an RSF1-Duet expression vector (Novagen) fused to an N-terminal 14-histidine tag. The protein was expressed by 1 mM isopropyl-D-thiogalactoside induction in *E. coli* strain BL21(DE3) Rosetta2 grown overnight in LB (Lysogeny Broth) at 20 °C and purified by Ni-NTA and heparin chromatography (GE Healthcare), followed by TEV cleavage of the tag and size-exclusion chromatography. The purified DP1 protein was concentrated to 3 mg ml^−1^ in 20 mM Tris HCl pH 8, 200 mM NaCl, 5% glycerol. The purified DP2 protein was concentrated to 10 mg ml^−1^ in 20 mM Tris HCl (pH 7.5) and 50 mM NaCl. Both purified proteins were flash frozen in liquid nitrogen and stored at −80 °C.

### Biochemical 3′–5′ exonuclease and primer-elongation assays

#### Oligonucleotides

Nucleotide sequences of the DNA substrates were inspired by Jokela *et al*.^24^. A 27mer DNA primer with three non-complementary bases at its 3′-end was used in the exonuclease assays (5′-ACGCCAGGCTTCGCCAGTCACGAT**ACT**-3′). A 24mer DNA primer was used in the DNAP primer-extension assays (5′-ACGCCAGGCTTCGCCAGTCACGAC-3′). The same 60mer DNA template was used in both reactions (5′-GCGGACTGCGATCGTACCTACGGACCTGCAGCTGACGTCGTGACTGGCGAAGCCTGGCGT-3′).

#### ^32^P-labelling

DNA primers were 5′-labelled with ^32^P using T4 polynucleotide kinase (PNK). A unit of 20 μM DNA was incubated for 1 h at 37 °C with 10 U PNK (Thermo scientific) and 100 μCi γ-^32^P-labelled ATP. The labelling reaction was stopped by heating the sample at 70 °C for 10 min.

#### DNA duplex annealing

A unit of 40 μM template was mixed with 40 μM labelled primer, annealing buffer (20 mM Tris-HCl (pH 8), 10 mM MgCl_2_ and 1 mM EDTA) and incubated for 5 min at 95 °C.

#### Activity tests

DP1: 10 μM DP1(144–622), 50 nM DNA duplex, 25 mM Tris-HCl pH7.6, 25 mM NaCl and 2 mM MgCl_2_ were incubated at 55 °C for 10, 45, 90 and 150 s. DP2: 50 nM DNA duplex was incubated at 55 °C with 25, 35 or 50 μM DP2(1–1,061) for 1, 5 or 10 min, in presence of 200 nM dNTPs and NEB 2.1 reaction buffer (New England Biolabs). All reactions were stopped by adding formamide. Samples were run through a 15% acrylamide–8 M urea sequencing gel and revealed using a PhosphorImager Storm 860 (Fujifilm).

### Crystallization

DP1 was crystallized by hanging-drop vapour diffusion at 4 °C, mixing 1 μl of protein (3 mg ml^−1^) with 1 μl of crystallization buffer containing 100 mM sodium cacodylate, pH 6.7, 200 mM calcium acetate and 2–6% PEG 8000. Crystals of DP1 bound to dAMP were obtained by co-crystallization in presence of 5 mM dAMP. DP2 was crystallized by sitting-drop vapour diffusion at 18 °C, mixing 1 μl of protein (10 mg ml^−1^) with 1 μl of crystallization buffer containing 60 mM MES, pH 5.6, 300 mM sodium chloride and 6% PEG 6000. Both DP1 and DP2 crystals were optimized using micro-seeding from a solution of crushed crystals. DP1 and DP2 crystals were cryo-cooled with 35% and 27.5% ethylene glycol, respectively. For the purpose of phasing (see below), DP1 and DP2 crystals were soaked overnight in a solution containing 5 mM K_2_Pt(NO_2_)_4_ (Jena Bioscience).

### Phasing and structure determination

*DP1*. Platinum-derived crystals diffracted up to 2.8 Å on Proxima 2 (SOLEIL, Gif-sur-Yvette, France). The dAMP-bound DP1 crystals diffracted up to 2.5 Å on ID23-1 (ESRF, Grenoble, France). The crystal structure was determined with phase information using PHENIX^57^ derived from anomalous scattering data collected at the Pt L-III edge (eight sites, figure-of-merit 0.45). The crystals belonged to the P2_1_2_1_2_1_ space group with two copies of the protein in the asymmetric unit. An initial model was obtained using PHENIX^57^, completed manually in Coot^58^ and refined in Buster^59^. For details, see Table 1. DP2: platinum-derived crystals diffracted up to 3.1 Å on Proxima 1 (SOLEIL, Gif-sur-Yvette, France). Native DP2 crystals diffracted up to 2.2 Å on ID23-1 (ESRF, Grenoble, France). Initial phases were derived using PHENIX^57^ from anomalous scattering data collected at the Pt L-III edge (nine sites, figure-of-merit 0.40). The crystal structure of the NTD (48–291) of *P. horikoshii* DP2 subunit (PDBid: 3,059 (ref. 10)) was fitted in the electron density on superposition to the initial model generated by AutoBuild^57^. Subsequent rounds of phase combination and automatic model building were notably improved. The crystals belong to the P2_1_2_1_2_1_ space group with one copy of the protein in the asymmetric unit. The model was then built manually in Coot^58^. For details, see Table 1.

### Model building and data refinement

*DP1*. The final model was refined in Buster to *R*/*R*_free_ values of 19.7/22.5 at 2.5 Å resolution. Non-crystallographic symmetry restraints were used throughout refinement, with one TLS parameter per molecule. Residues 144–151, 164–173, 213–224 and 514–517 (only in chain B), which were not visible in the electron density, were not included in the final model. One dAMP molecule and 66 water molecules were added per monomer. In the final electron density, remaining peaks that could not be attributed to water were modelled with acetate, ethylene glycol and three calcium ions, which were present in the crystallization solution (see above). The active site includes two tightly bound metal ions, zinc and iron, whose presence was confirmed by collecting anomalous data at the zinc and iron K-edges (see below and Supplementary Fig. 2). Of all residues, 96.2% were in the favoured regions of the Ramachandran plot with three outliers. Two out of these three outliers correspond to His residues that participate to metal ion coordination and are located in the active site in a region where the electron density is clearly defined. Metal ion coordination causes a subtle distortion of the backbone resulting with these residues being Ramachandran outliers. The Molprobity^60^ score for the refined model is 1.60, in the 99th percentile of structures refined at comparable resolution. DP2: the final model was refined in Buster to *R*/*R*_free_ values of 19.9/23.5 at 2.2 Å resolution, with five TLS parameters (defined using the TLSMD^61^ web server) for the protein chain. Residues 1–3, 286–307, 325–338, 358–363, 376–392, 654–667, 1,000–1,010 and 1,041–1,061, which were not visible in the electron density were not included in the final model. Those residues are concentrated in a region that connects the NTD to the central domain. These residues are highly divergent between PolDs from different species. To gain confidence in the assignment of the sequence of the protein within regions where the chain is not continuous, we collected an additional anomalous data set at the sulphur edge (1.8 Å). Taking advantage of the 10 cysteines and 14 methionines of the protein, we confirmed the correctness of sequence assignment. In all, 242 water molecules and 2 zinc ions (see below and Supplementary Fig. 7) were added to the model. Of all residues, 97.4% were in the favoured regions of the Ramachandran plot with no outliers. The Molprobity^60^ score for the refined model is 1.43, in the 99th percentile of structures refined at comparable resolution. The quality of the electron density is illustrated in Supplementary Fig. 10.

### Assigning transition metal ions identities

*DP1*. The active site of DP1 contains His, Asp and Asn residues, which are conserved within the phosphodiesterase protein superfamily and coordinate tightly two catalytic metal ions. The Fourier Fo–Fc difference electron density map shows two strong peaks at 12*σ* in the active site of DP1, consistent with the presence of two metal ions. An X-ray fluorescence scan on DP1 crystals detected the presence of three transition metal ions: zinc, iron and calcium. While calcium is present in the crystallization solution (see above), neither iron nor zinc ions were introduced in purification or crystallization buffers. To assign the identity of the two metal ions in the active site of DP1 (arbitrarily named sites A and B), we collected four anomalous data sets: the first one at the iron K-edge (1.7394 Å), the second one right below the iron K-edge (1.7557 Å), the third one at the zinc K-edge (1.2815 Å) and the fourth one right below the zinc K-edge (1.2881 Å) (Supplementary Fig. 2). All data sets were collected on different regions of the same crystal. The anomalous map calculated from the data set collected at the iron K-edge shows two peaks: a stronger peak at site A (10.5*σ*), a weaker peak at site B (7.6*σ*). The peak at site A is lost in the anomalous map calculated from the data set collected below the iron K-edge, thereby showing that iron ions specifically binds at site A. The anomalous map calculated from the dataset collected at the zinc K-edge shows two closely equivalent peaks at site A (10.2*σ*) and at site B (8.9*σ*). Both peaks are substantially lower in the anomalous map calculated from the data set collected below the zinc K-edge (5.1*σ*), suggesting that zinc ions bind to both sites. Overall, this set of experiments shows that iron ion binds mainly to site A, zinc ions to both A and B sites. In the final model, site A and B were modelled with iron and zinc ions, respectively, in line with other calcineurin-like phosphodiesterases, which show dual binding of zinc and iron ions within their active site: for example, mammalian calcineurin^15^ (PDBid 1TCO) and the red kidney bean purple acid phosphatase^16^ (PDBid 1KBP). DP2: the final model includes two metal-binding motifs, each coordinated by four cysteine ligands. To confirm the presence of zinc within these motifs, we collected two anomalous data sets: the first one at the zinc K-edge (1.2820 Å), the second one right below the zinc K-edge (1.2882 Å). While the anomalous map calculated from the data set collected at the zinc K-edge shows two strong peaks (17*σ* for zinc I, 9*σ* for zinc II), the anomalous map calculated from the data set collected below the zinc K-edge shows no such peaks, thereby confirming the presence of zinc in both sites (Supplementary Fig. 8).

### Structure analysis

Dali^25^ was used to compare the DP1 and DP2 structures with those of the Protein Data Bank. The programme Superpose implemented in CCP4 (ref. 62) was used for structural alignments and Areaimol^63^ for accessible surface area measurements (CCP4). Electrostatic surface potentials were calculated using APBS^64^ in Chimera^65^. The evolutionary conservation analysis of surface residues was performed with the Consurf^66^ server. Figures were prepared with Chimera^65^ and PyMOL (The PyMOL Molecular Graphics System, Version 1.8 Schrödinger, LLC.).

### Data availability

Coordinates and structure factors for DP1 and DP2 crystal structures were deposited in the Protein Data Bank under the accession codes 5IHE and 5IJL, respectively. All other data are available in the manuscript and associated materials, or from the authors on reasonable request.

## Additional information

**How to cite this article:** Sauguet, L. *et al*. Shared active site architecture between archaeal PolD and multi-subunit RNA polymerases revealed by X-ray crystallography. *Nat. Commun.* 7:12227 doi: 10.1038/ncomms12227 (2016).

## Supplementary Material

Supplementary Information Supplementary Figures 1-10, and Supplementary References.

## Figures and Tables

**Table 1:** Data collection and refinement statistics.

	**DP1 Pt-derivative**	**DP1+dAMP**	**DP2 Pt-derivative**	**DP2 native**
*Data collection*				
Space group	P2_1_2_1_2_1_	P2_1_2_1_2_1_	P2_1_2_1_2_1_	P2_1_2_1_2_1_
Wavelength (Å)	1.0715	0.9919	1.0718	0.9840
Cell dimensions				
*a*, *b*, *c* (Å)	85.9, 91.6, 143.6	85.9, 91.4, 145.2	103.8, 106.2, 106.2	103.8, 106.2, 110.9
*α*, *β*, *γ* (°)	90, 90, 90	90, 90, 90	90, 90, 90	90, 90, 90
Resolution (Å)	47.9–2.81 (2.96–2.81)*	48.3–2.50 (2.64–2.50)	49.4–3.12 (3.34–3.12)	43.8–2.19 (2.32–2.19)
Rsym (%)	3.3 (19.4)	6.1 (82.5)	6.3 (34.6)	7.9 (115.1)
*I*/*σ*(*I*)	23.6 (4.2)	6.9 (1.1)	15.5 (3.5)	8.5 (1.01)
Completeness (%)	99.7 (98.0)	99.3 (99.7)	99.3 (96.1)	98.9 (94.2)
Redundancy	14.5 (14.4)	4.5 (4.4)	8.6 (8.7)	5.5 (4.9)
CC_(1/2)_† (%)	99.6 (72.7)	98.9 (50.6)	99.6 (72.6)	99.6 (44.0)
				
*Refinement*				
Resolution (Å)		47.2–2.50		43.8–2.19
No. of reflections		39,885		63,302
*R*_work_*/R*_free_		19.7/22.5		19.9/23.5
No. of atoms				
Protein		7,050		7,525
Ligand		59		
Metal ion		7		2
Water		66		242
B-factors (Å^2^)				
Protein		86.1		53.4
Ligand		91.3		
Metal ion		84.2		85.8
Water		69.5		50.8
R.m.s.d.				
Bond lengths (Å)		0.008		0.010
Bond angles (°)		1.08		1.09
Ramachandran				
Favoured/outliers (%)‡		96.2/0.35		97.4/0.0
Molprobity^16^ score		99th		99th

^*^Numbers in parenthesis refer to the highest-resolution shell.

^†^CC_(1/2)_=percentage of correlation between intensities from random half-data sets^17^.

^‡^Calculated with MolProbity^16^.

## References

[b1] Kornberg A. & Baker T. A. DNA Replication University Science Books (2005).

[b2] Patel P. H. & Loeb L. A. Getting a grip on how DNA polymerases function. Nat. Struct. Biol. 8, 656–659 (2001). 11473246 10.1038/90344

[b3] Braithwaite D. K. & Ito J. Compilation, alignment, and phylogenetic relationships of DNA polymerases. Nucleic Acids Res. 21, 787–802 (1993). 8451181 10.1093/nar/21.4.787 PMC309208

[b4] Wu S., Beard W. A., Pedersen L. G. & Wilson S. H. Structural comparison of DNA polymerase architecture suggest a nucleotide gateway to the polymerase active site. Chem. Rev. 114, 2759–2774 (2014). 24359247 10.1021/cr3005179 PMC4017774

[b5] Cann I. K., Komori K., Toh H., Kanai S. & Ishino Y. A heterodimeric DNA polymerase: evidence that members of Euryarchaeota possess a distinct DNA polymerase. Proc. Natl Acad. Sci. USA 95, 14250–14255 (1998). 9826686 10.1073/pnas.95.24.14250 PMC24359

[b6] Greenough L., Kelman Z. & Gardner A. F. The roles of family B and D DNA polymerases in Thermococcus species 9°N Okazaki fragment maturation. J. Biol. Chem. 290, 12514–12522 (2015). 25814667 10.1074/jbc.M115.638130 PMC4432273

[b7] Henneke G., Flament D., Hübscher U., Querellou J. & Raffin J.-P. The hyperthermophilic euryarchaeota *Pyrococcus abyssi* likely requires the two DNA polymerases D and B for DNA replication. J. Mol. Biol. 350, 53–64 (2005). 15922358 10.1016/j.jmb.2005.04.042

[b8] Cubonová L. . Archaeal DNA polymerase D but not DNA polymerase B is required for genome replication in *Thermococcus kodakarensis*. J. Bacteriol. 195, 2322–2328 (2013). 23504010 10.1128/JB.02037-12 PMC3650531

[b9] Yamasaki K., Urushibata Y., Yamasaki T., Arisaka F. & Matsui I. Solution structure of the N-terminal domain of the archaeal D-family DNA polymerase small subunit reveals evolutionary relationship to eukaryotic B-family polymerases. FEBS Lett. 584, 3370–3375 (2010). 20598295 10.1016/j.febslet.2010.06.026

[b10] Matsui I., Urushibata Y., Shen Y., Matsui E. & Yokoyama H. Novel structure of an N-terminal domain that is crucial for the dimeric assembly and DNA-binding of an archaeal DNA polymerase D large subunit from *Pyrococcus horikoshii*. FEBS Lett. 585, 452–458 (2011). 21192935 10.1016/j.febslet.2010.12.040

[b11] Aravind L. & Koonin E. V. Phosphoesterase domains associated with DNA polymerases of diverse origins. Nucleic Acids Res. 26, 3746–3752 (1998). 9685491 10.1093/nar/26.16.3746 PMC147763

[b12] Tahirov T. H., Makarova K. S., Rogozin I. B., Pavlov Y. I. & Koonin E. V. Evolution of DNA polymerases: an inactivated polymerase-exonuclease module in Pol ɛ and a chimeric origin of eukaryotic polymerases from two classes of archaeal ancestors. Biol. Direct 4, 11 (2009). 19296856 10.1186/1745-6150-4-11 PMC2669801

[b13] Shen Y., Tang X.-F., Yokoyama H., Matsui E. & Matsui I. A 21-amino acid peptide from the cysteine cluster II of the family D DNA polymerase from *Pyrococcus horikoshii* stimulates its nuclease activity which is Mre11-like and prefers manganese ion as the cofactor. Nucleic Acids Res. 32, 158–168 (2004). 14704353 10.1093/nar/gkh153 PMC373266

[b14] Tang X.-F., Shen Y., Matsui E. & Matsui I. Domain topology of the DNA polymerase D complex from a hyperthermophilic archaeon *Pyrococcus horikoshii*. Biochemistry 43, 11818–11827 (2004). 15362867 10.1021/bi0362931

[b15] Griffith J. P. . X-ray structure of calcineurin inhibited by the immunophilin-immunosuppressant FKBP12-FK506 complex. Cell 82, 507–522 (1995). 7543369 10.1016/0092-8674(95)90439-5

[b16] Sträter N., Klabunde T., Tucker P., Witzel H. & Krebs B. Crystal structure of a purple acid phosphatase containing a dinuclear Fe(III)-Zn(II) active site. Science 268, 1489–1492 (1995). 7770774 10.1126/science.7770774

[b17] Yu L., Golbeck J., Yao J. & Rusnak F. Spectroscopic and enzymatic characterization of the active site dinuclear metal center of calcineurin: implications for a mechanistic role. Biochemistry 36, 10727–10734 (1997). 9271503 10.1021/bi970519g

[b18] Paull T. T. & Gellert M. The 3′ to 5′ exonuclease activity of Mre 11 facilitates repair of DNA double-strand breaks. Mol. Cell 1, 969–979 (1998). 9651580 10.1016/s1097-2765(00)80097-0

[b19] Hopfner K. P. . Structural biochemistry and interaction architecture of the DNA double-strand break repair Mre11 nuclease and Rad50-ATPase. Cell 105, 473–485 (2001). 11371344 10.1016/s0092-8674(01)00335-x

[b20] Bressan D. A., Olivares H. A., Nelms B. E. & Petrini J. H. Alteration of N-terminal phosphoesterase signature motifs inactivates *Saccharomyces cerevisiae* Mre11. Genetics 150, 591–600 (1998). 9755192 10.1093/genetics/150.2.591 PMC1460356

[b21] Palud A. . Intrinsic properties of the two replicative DNA polymerases of *Pyrococcus abyssi* in replicating abasic sites: possible role in DNA damage tolerance? Mol. Microbiol. 70, 746–761 (2008). 18826407 10.1111/j.1365-2958.2008.06446.x

[b22] Rusnak F. & Mertz P. Calcineurin: form and function. Physiol. Rev. 80, 1483–1521 (2000). 11015619 10.1152/physrev.2000.80.4.1483

[b23] de Jager M., Wyman C., van Gent D. C. & Kanaar R. DNA end-binding specificity of human Rad50/Mre11 is influenced by ATP. Nucleic Acids Res. 30, 4425–4431 (2002). 12384589 10.1093/nar/gkf574 PMC137138

[b24] Jokela M., Eskelinen A., Pospiech H., Rouvinen J. & Syväoja J. E. Characterization of the 3′ exonuclease subunit DP1 of *Methanococcus jannaschii* replicative DNA polymerase D. Nucleic Acids Res. 32, 2430–2440 (2004). 15121900 10.1093/nar/gkh558 PMC419447

[b25] Holm L. & Rosenström P. Dali server: conservation mapping in 3D. Nucleic Acids Res. 38, W545–W549 (2010). 20457744 10.1093/nar/gkq366 PMC2896194

[b26] Baranovskiy A. G. . X-ray structure of the complex of regulatory subunits of human DNA polymerase δ. Cell Cycle 7, 3026–3036 (2008). 18818516 10.4161/cc.7.19.6720 PMC2605013

[b27] Suwa Y. . Crystal structure of the human Pol α B subunit in complex with the C-terminal domain of the catalytic subunit. J. Biol. Chem. 290, 14328–14337 (2015). 25847248 10.1074/jbc.M115.649954 PMC4505502

[b28] Delarue M. . Crystal structure of a prokaryotic aspartyl tRNA-synthetase. EMBO J. 13, 3219–3229 (1994). 8045252 10.1002/j.1460-2075.1994.tb06623.x PMC395218

[b29] Shamoo Y., Friedman A. M., Parsons M. R., Konigsberg W. H. & Steitz T. A. Crystal structure of a replication fork single-stranded DNA binding protein (T4 gp32) complexed to DNA. Nature 376, 362–366 (1995). 7630406 10.1038/376362a0

[b30] Arcus V. OB-fold domains: a snapshot of the evolution of sequence, structure and function. Curr. Opin. Struct. Biol. 12, 794–801 (2002). 12504685 10.1016/s0959-440x(02)00392-5

[b31] Makarova K. S., Krupovic M. & Koonin E. V. Evolution of replicative DNA polymerases in archaea and their contributions to the eukaryotic replication machinery. Front. Microbiol. 5, 354 (2014). 25101062 10.3389/fmicb.2014.00354 PMC4104785

[b32] Shen Y. . Invariant Asp-1122 and Asp-1124 are essential residues for polymerization catalysis of family D DNA polymerase from *Pyrococcus horikoshii*. J. Biol. Chem. 276, 27376–27383 (2001). 11319225 10.1074/jbc.M011762200

[b33] Okuda M. . A novel zinc finger structure in the large subunit of human general transcription factor TFIIE. J. Biol. Chem. 279, 51395–51403 (2004). 15385556 10.1074/jbc.M404722200

[b34] Kelley L. A., Mezulis S., Yates C. M., Wass M. N. & Sternberg M. J. E. The Phyre2 web portal for protein modeling, prediction and analysis. Nat. Protoc. 10, 845–858 (2015). 25950237 10.1038/nprot.2015.053 PMC5298202

[b35] Ruprich-Robert G. & Thuriaux P. Non-canonical DNA transcription enzymes and the conservation of two-barrel RNA polymerases. Nucleic Acids Res. 38, 4559–4569 (2010). 20360047 10.1093/nar/gkq201 PMC2919709

[b36] Iyer L. M., Koonin E. V. & Aravind L. Evolutionary connection between the catalytic subunits of DNA-dependent RNA polymerases and eukaryotic RNA-dependent RNA polymerases and the origin of RNA polymerases. BMC Struct. Biol. 3, 1 (2003). 12553882 10.1186/1472-6807-3-1 PMC151600

[b37] Werner F. & Grohmann D. Evolution of multisubunit RNA polymerases in the three domains of life. Nat. Rev. Microbiol. 9, 85–98 (2011). 21233849 10.1038/nrmicro2507

[b38] Lehmann E., Brueckner F. & Cramer P. Molecular basis of RNA-dependent RNA polymerase II activity. Nature 450, 445–449 (2007). 18004386 10.1038/nature06290

[b39] Sainsbury S., Niesser J. & Cramer P. Structure and function of the initially transcribing RNA polymerase II-TFIIB complex. Nature 493, 437–440 (2013). 23151482 10.1038/nature11715

[b40] Makeyev E. V. & Bamford D. H. Cellular RNA-dependent RNA polymerase involved in posttranscriptional gene silencing has two distinct activity modes. Mol. Cell 10, 1417–1427 (2002). 12504016 10.1016/s1097-2765(02)00780-3

[b41] Salgado P. S. . The structure of an RNAi polymerase links RNA silencing and transcription. PLoS Biol. 4, e434 (2006). 17147473 10.1371/journal.pbio.0040434 PMC1750930

[b42] Castrec B., Laurent S., Henneke G., Flament D. & Raffin J.-P. The glycine-rich motif of *Pyrococcus abyssi* DNA polymerase D is critical for protein stability. J. Mol. Biol. 396, 840–848 (2010). 20070946 10.1016/j.jmb.2010.01.006

[b43] Cann I. K. & Ishino Y. Archaeal DNA replication: identifying the pieces to solve a puzzle. Genetics 152, 1249–1267 (1999). 10430556 10.1093/genetics/152.4.1249 PMC1460685

[b44] Castrec B. . Binding to PCNA in euryarchaeal DNA replication requires two PIP motifs for DNA polymerase D and one PIP motif for DNA polymerase B. J. Mol. Biol. 394, 209–218 (2009). 19781553 10.1016/j.jmb.2009.09.044

[b45] Ollis D. L., Brick P., Hamlin R., Xuong N. G. & Steitz T. A. Structure of large fragment of *Escherichia col*i DNA polymerase I complexed with dTMP. Nature 313, 762–766 (1985). 3883192 10.1038/313762a0

[b46] Beese L. S., Derbyshire V. & Steitz T. A. Structure of DNA polymerase I Klenow fragment bound to duplex DNA. Science 260, 352–355 (1993). 8469987 10.1126/science.8469987

[b47] Delarue M., Poch O., Tordo N., Moras D. & Argos P. An attempt to unify the structure of polymerases. Protein Eng. 3, 461–467 (1990). 2196557 10.1093/protein/3.6.461

[b48] Lamers M. H., Georgescu R. E., Lee S.-G., O'Donnell M. & Kuriyan J. Crystal structure of the catalytic alpha subunit of *E. coli* replicative DNA polymerase III. Cell 126, 881–892 (2006). 16959568 10.1016/j.cell.2006.07.028

[b49] Bailey S., Wing R. A. & Steitz T. A. The structure of *T. aquaticus* DNA polymerase III is distinct from eukaryotic replicative DNA polymerases. Cell 126, 893–904 (2006). 16959569 10.1016/j.cell.2006.07.027

[b50] Evans R. J. . Structure of PolC reveals unique DNA binding and fidelity determinants. Proc. Natl Acad. Sci. USA 105, 20695–20700 (2008). 19106298 10.1073/pnas.0809989106 PMC2634937

[b51] Hamdan S., Carr P. D., Brown S. E., Ollis D. L. & Dixon N. E. Structural basis for proofreading during replication of the *Escherichia coli* chromosome. Structure 10, 535–546 (2002). 11937058 10.1016/s0969-2126(02)00738-4

[b52] Brautigam C. A. & Steitz T. A. Structural principles for the inhibition of the 3′-5′ exonuclease activity of *Escherichia coli* DNA polymerase I by phosphorothioates. J. Mol. Biol. 277, 363–377 (1998). 9514742 10.1006/jmbi.1997.1586

[b53] Wang J., Yu P., Lin T. C., Konigsberg W. H. & Steitz T. A. Crystal structures of an NH2-terminal fragment of T4 DNA polymerase and its complexes with single-stranded DNA and with divalent metal ions. Biochemistry 35, 8110–8119 (1996). 8679562 10.1021/bi960178r

[b54] Koonin E. V. Conserved sequence pattern in a wide variety of phosphoesterases. Protein Sci. 3, 356–358 (1994). 8003970 10.1002/pro.5560030218 PMC2142799

[b55] Schenk G. . Binuclear metallohydrolases: complex mechanistic strategies for a simple chemical reaction. Acc. Chem. Res. 45, 1593–1603 (2012). 22698580 10.1021/ar300067g

[b56] Killelea T., Ralec C., Bossé A. & Henneke G. PCR performance of a thermostable heterodimeric archaeal DNA polymerase. Front. Microbiol. 5, 195 (2014). 24847315 10.3389/fmicb.2014.00195 PMC4019886

[b57] Adams P. D. . The Phenix software for automated determination of macromolecular structures. Methods 55, 94–106 (2011). 21821126 10.1016/j.ymeth.2011.07.005 PMC3193589

[b58] Emsley P. & Cowtan K. Coot: model-building tools for molecular graphics. Acta Crystallogr. D Biol. Crystallogr. 60, 2126–2132 (2004). 15572765 10.1107/S0907444904019158

[b59] Blanc E. . Refinement of severely incomplete structures with maximum likelihood in BUSTER-TNT. Acta Crystallogr. D Biol. Crystallogr. 60, 2210–2221 (2004). 15572774 10.1107/S0907444904016427

[b60] Davis I. W. . MolProbity: all-atom contacts and structure validation for proteins and nucleic acids. Nucleic Acids Res. 35, W375–W383 (2007). 17452350 10.1093/nar/gkm216 PMC1933162

[b61] Painter J. & Merritt E. A. Optimal description of a protein structure in terms of multiple groups undergoing TLS motion. Acta Crystallogr. D Biol. Crystallogr. 62, 439–450 (2006). 16552146 10.1107/S0907444906005270

[b62] Krissinel E. & Henrick K. Secondary-structure matching (SSM), a new tool for fast protein structure alignment in three dimensions. Acta Crystallogr. D Biol. Crystallogr. 60, 2256–2268 (2004). 15572779 10.1107/S0907444904026460

[b63] Lee B. & Richards F. M. The interpretation of protein structures: estimation of static accessibility. J. Mol. Biol. 55, 379–400 (1971). 5551392 10.1016/0022-2836(71)90324-x

[b64] Baker N. A., Sept D., Joseph S., Holst M. J. & McCammon J. A. Electrostatics of nanosystems: application to microtubules and the ribosome. Proc. Natl Acad. Sci. USA 98, 10037–10041 (2001). 11517324 10.1073/pnas.181342398 PMC56910

[b65] Pettersen E. F. . UCSF Chimera—a visualization system for exploratory research and analysis. J. Comput. Chem. 25, 1605–1612 (2004). 15264254 10.1002/jcc.20084

[b66] Landau M. . ConSurf 2005: the projection of evolutionary conservation scores of residues on protein structures. Nucleic Acids Res. 33, W299–W302 (2005). 15980475 10.1093/nar/gki370 PMC1160131

[b67] Park Y. B., Chae J., Kim Y. C. & Cho Y. Crystal structure of human Mre11: understanding tumorigenic mutations. Structure 19, 1591–1602 (2011). 22078559 10.1016/j.str.2011.09.010

[b68] Schiller C. B. . Structure of Mre11-Nbs1 complex yields insights into ataxia-telangiectasia-like disease mutations and DNA damage signaling. Nat. Struct. Mol. Biol. 19, 693–700 (2012). 22705791 10.1038/nsmb.2323 PMC3392456

[b69] Möckel C., Lammens K., Schele A. & Hopfner K.-P. ATP driven structural changes of the bacterial Mre11:Rad50 catalytic head complex. Nucleic Acids Res. 40, 914–927 (2012). 21937514 10.1093/nar/gkr749 PMC3258140

[b70] Klinge S., Núñez-Ramírez R., Llorca O. & Pellegrini L. 3D architecture of DNA Pol alpha reveals the functional core of multi-subunit replicative polymerases. EMBO J. 28, 1978–1987 (2009). 19494830 10.1038/emboj.2009.150 PMC2693882

