# Supplementary material for: Shared active site architecture between archaeal PolD and multi-subunit RNA polymerases revealed by X-ray crystallography
Source: Nat Commun. 2016 Aug 22;0:12227. doi: 10.1038/ncomms12227 (PMC4996933; doi:10.1038/ncomms12227)
Supplement: Supplementary Information — Supplementary Figures 1-10, and Supplementary References. [file ncomms12227-s1.pdf]

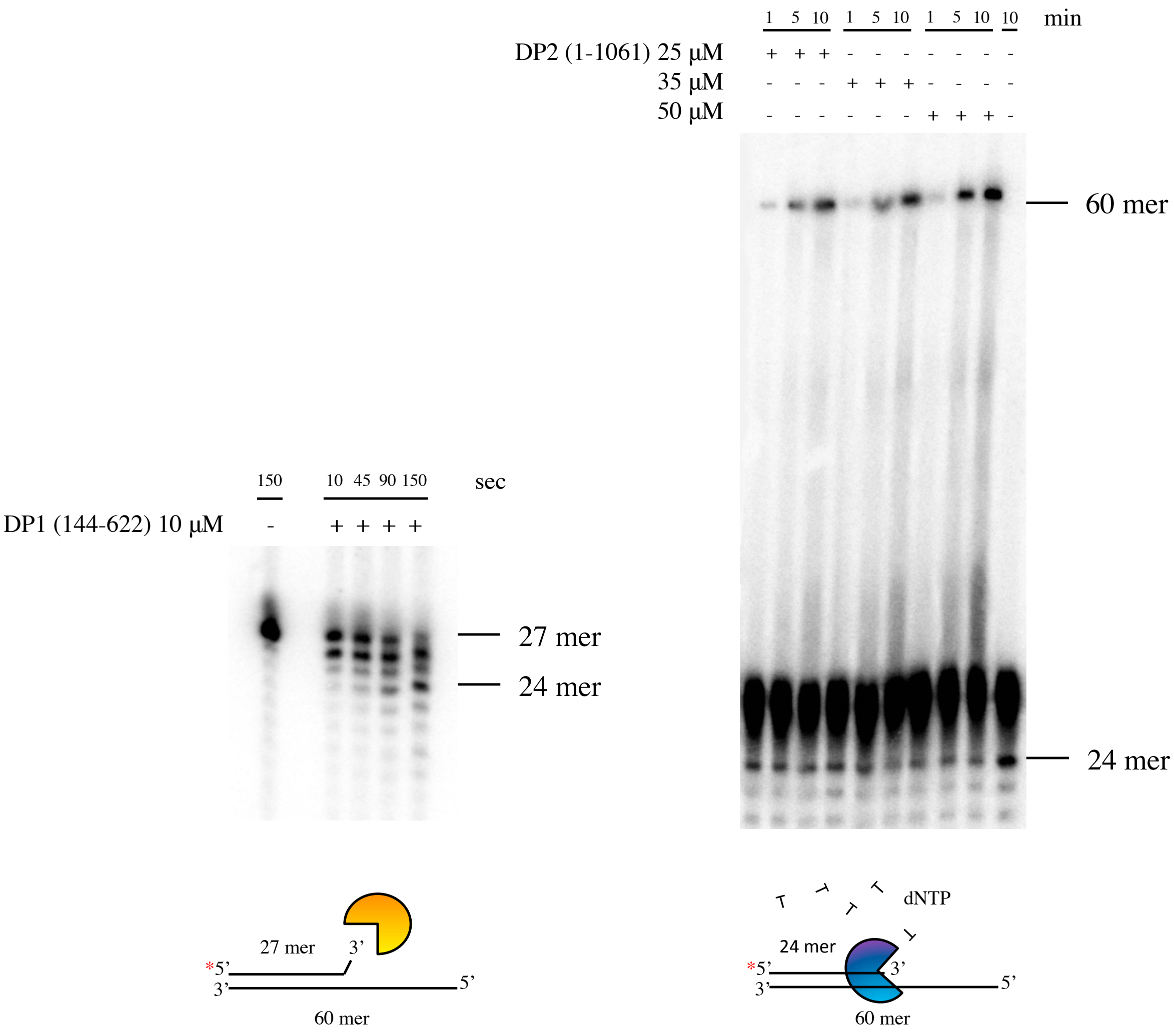

Supplementary Figure 1: DP1(144-622) possesses 3’-5’ exonuclease activity and DP2(1-1061) is capable of 5’-3’ DNA primer extension. Left panel shows the time-course of the 3’-5’ digestion of mispaired nucleotides by DP1(144-622). A control experiment obtained in absence of DP1 is also shown (left band). Right panel shows time-course of the 5’-3’ primer-elongation assays with the indicated increasing amounts of DP2(1-1061). A control experiment was performed in absence of DP2 (right band). Details for the nuclease and primer-extension activity assays are provided in Methods section.

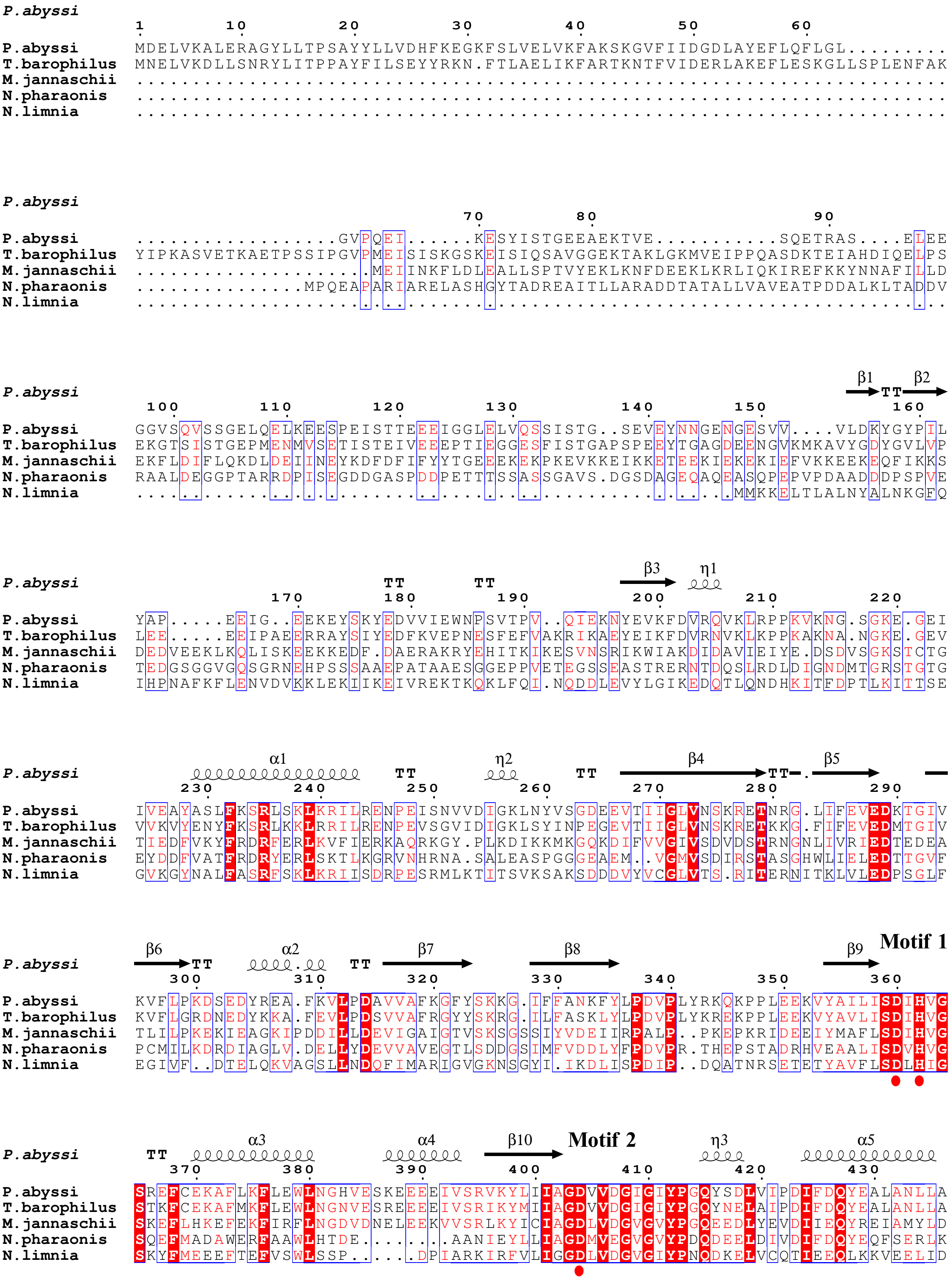
P.abyssi
1 10 20 30 40 50 60
P.abyssi MDELVKALERAGYLLTPSAYYLLVDHFKEGKFSLVELVKFAKSKGVFIIDGDLAYEFLQFLGL.........
T.barophilus MNELVKDLLSNRYLITPPAYFILSEYYRKN.FTLAELIKFARTKNTFVIDERLAKEFLESKGLLSPLENFAK
M.jannaschii ....
N.pharaonis ....
N.limnia ....
P.abyssi
70 80 90
P.abyssi ..................GVPQEI......KESYISTGEEAEKTVE..........SQETRAS....ELEE
T.barophilus YIPKASVETKAETPSSIPGVPMEISISKGSKEISIQSAVGGEKTAKLGKMVEIPPQASDKTEIAHDIQELPS
M.jannaschii .....................MEIINKFLDLEALLSPTVYEKLKNFDEEKLKRLIQKIREFKKYNNAFILLD
N.pharaonis ...............MPQEAPARIARELASHGYTADREAITLLARADDTATALLVAVEATPDDALKLTADDV
N.limnia ....
P.abyssi
β1 β2
TT
100 110 120 130 140 150 160
P.abyssi GGVSQVSSGELQELKEESPEISTTEEEIGGLELVQSSISTG..SEVEYNNGENGESVV....VLDKYGYPIL
T.barophilus EKGTSISTGEPMENMVSETISTEIVEEEPTIEGGESFISTGAPSPEEYTGAGDEENGVKMKAVYGDYGVLVP
M.jannaschii EKFLDIFLQKDLDEIINEYKDFDFIFYYTGEEEKEKPKEVKKEIKKETEEKIEKEKIEFVKKEEKEQFIKKS
N.pharaonis RAALDEGGPTARRDPISEGDDGASPDDPETTTSSASSGAVS.DGSDAGEQAQEASQPEPVPDAADDDPSPVE
N.limnia ....MMKKELTLALNYALNKGFQ
P.abyssi
β3 η1
TT TT
170 180 190 200 210 220
P.abyssi YAP.....EEIG..EEKEYSKYEDVVIEWNPSVTPV..QIEKNYEVKFDVRQVKLRPPKVKNG.SGKE.GEI
T.barophilus LEE.....EEIPAEERRAYSIYEDFKVEPNESFEFVAKRIKAEYEIKFDVRNVKLKPPKAKNA.NGKE.GEV
M.jannaschii DEDVEEKLKQLISKEEKKEDF.DAERAKRYEHITKIKESVNSRIKWIAKDIDAVIEIYE.DSDVSGKSTCTG
N.pharaonis TEDGSGGVGQSGRNEHPSSSAAEPATAAESGGEPPVETEGSSEASTRERNTDQSLRDLDIGNDMTGRSTGTG
N.limnia IHPNAFKFLENVDVKKLEKIIKEIVREKTKQKLFQI.NQDDLEVYLGIKEDQTLQNDHKITFDPTLKITTSE
P.abyssi
α1 η2 β4 β5
TT TT TT
230 240 250 260 270 280 290
P.abyssi IVEAYASLFKSRLSKLKRILRENPEISNVVDIGKLNYVSGDEEVTIIGLVNSKRETNRG.LIFEVEDKTGIV
T.barophilus VVKVYENYFKSRLKKLRRILRENPEVSGVIDIGKLSYINPEGEVTIIGLVNSKRETKKG.FIFEVEDMTGIV
M.jannaschii TIEDFVKYFRDRFERLKVFIERKAQRKGY.PLKDIKKMKGQKDIFVVGIVSDVDSTRNGNLIVRIEDTEDEA
N.pharaonis EYDDFVATFRDRYERLSKTLKGRVNHRNA.SALEASPGGGEAEM..VGMVSDIRSTASGHWLIELEDTGVF
N.limnia GVKGYNALFASRFSKLKRIISDRPESRMLKTITSVKSAKSDDDVYVCGLVTS.RITERNITKLVLEDPSGLF
P.abyssi
β6 α2 β7 β8 β9 Motif 1
TT TT
300 310 320 330 340 350 360
P.abyssi KVFLPKDSEDYREA.FKVLPDAVVAFKGFYSKKG.IFFANKFYLPDVPLYRKQKPPLEEKVYAILISDIHVG
T.barophilus KVFLGRDNEDYKKA.FEVLPDSVVAFRGYYSKRG.ILFASKLYLPDVPLYKREKPPLEEKVYAVLISDIHVG
M.jannaschii TLILPKEKIEAGKIPDDILLDEVIGAIGTVSKSGSSIYVDEIIRPALP..PKEPKRIDEEIYMAFLSDIHVG
N.pharaonis PCMILKDRDIAGLV.DELLYDEVVAVEGTLSDDGSIMFVDDLYFPDVPR.THEPSTADRHVEAALISDVHVG
N.limnia EGIVF..DTELQKVAGSLLNDQFIMARIGVGKNSGYI.IKDLISPDIP..DQATNRSETETYAVFLSDLHIG
P.abyssi
α3 α4 β10 Motif 2 η3 α5
TT
370 380 390 400 410 420 430
P.abyssi SREFCEKAFLKFLEWLNGHVESKEEEEIVSRVKYLIIAGDVVDGIGIYPGQYSDLVIPDIFDQYEALANLLA
T.barophilus STKFCEKAFMKFLEWLNGNVESREEEEIVSRIKYMIIAGDVVDGIGIYPGQYNELAIPDIFDQYEALANLLA
M.jannaschii SKEFLHKEFEKFIRFLNGDVDNELEEKVVSRLKYICIAGDLVDGVGVYPGQEEDLYEVDIIEQYREIAMYLD
N.pharaonis SQEFMADAWERFAAWLHTDE........AANIEYLLIAGDMVEGVGVYPDQDEELDIVDIFDQYEQFSERLK
N.limnia SKYFMEEEFTEFVSWLSSP.......DPIARKIRFVLIGGDLVDGVGIYPNQDKELVCQTIEEQLKKVEELID

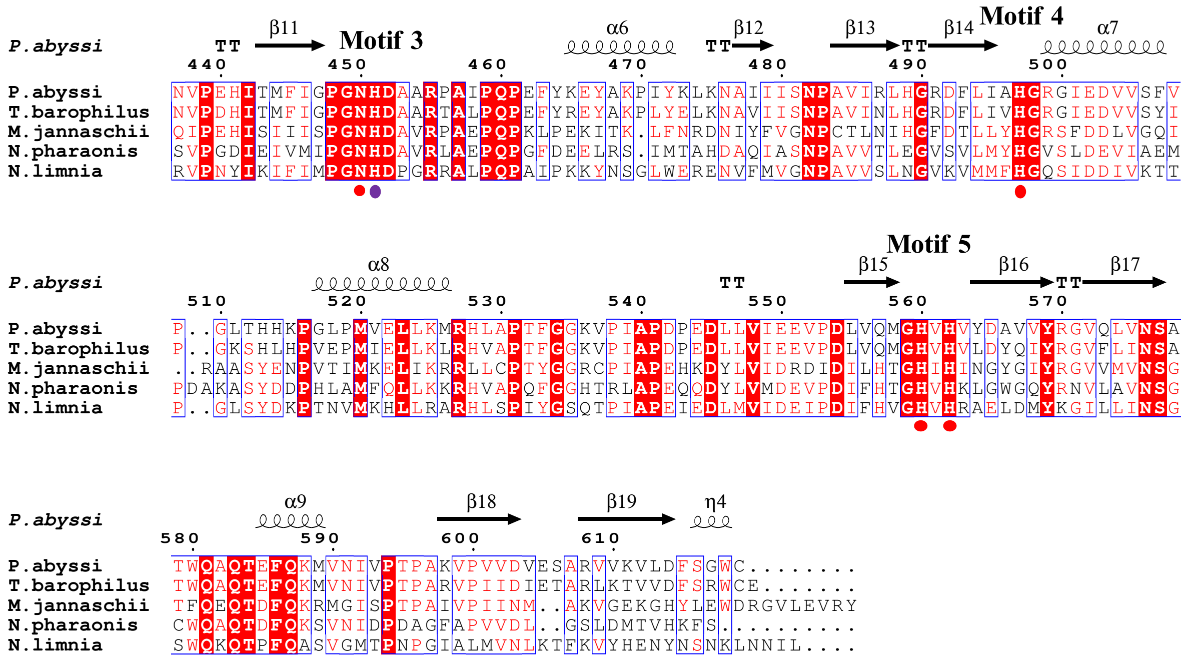

Supplementary Figure 2: Alignment of DP1 structure amino acid sequence with other DP1 subunits. MultAlin[1] and ESPript[2] 3.0 were used to generate an alignment with four representative protein sequences whose sequence diversity was chosen to best illustrate the sequence variability (GI accession number): *Pyrococcus abyssi* (499168969), *Thermococcus barophilus* (948739970), *Methanocadococcus jannaschii* (2833545), *Natronomonas pharaonis* (499641234), *Nitrosoarchaeum limnia* (329137458). Conserved residues are boxed in red and highly conserved residues are coloured in red. Secondary structure elements and phosphodiesterase conserved motifs observed in *P. abyssi* DP1 structure are also shown. Metal-coordinating residues and proton donor residues are outlined by red and purple circles, respectively.

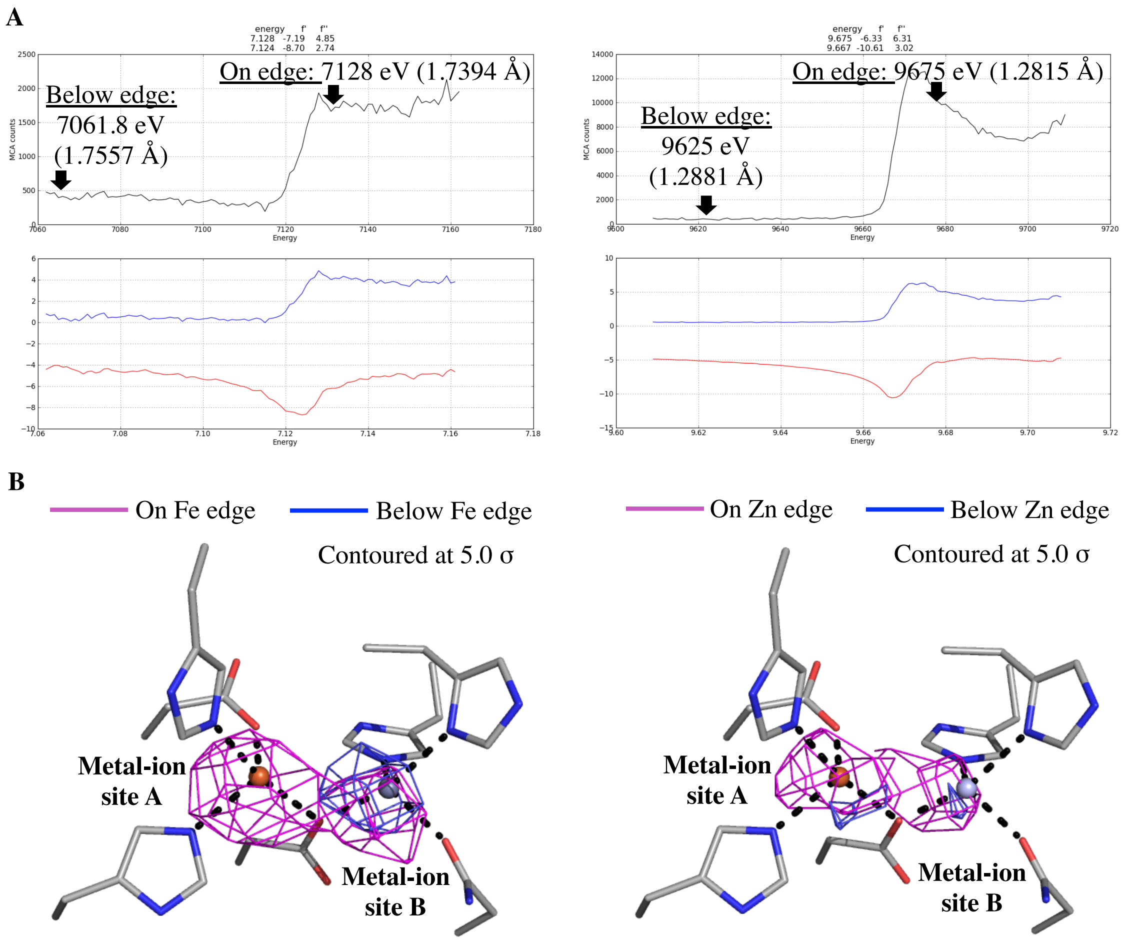

Supplementary Figure 3: DP1 contains a dinuclear Fe^{3+}-Zn^{2+} active site. Validation of metal-ion binding sites by comparing anomalous maps calculated from datasets collected at different energies. (A) Plots showing the anomalous scattering coefficients as a function of energy around the iron (left) and zinc (right) K-edges. (B) Close-view of the dinuclear Fe^{3+}-Zn^{2+} DP1 active site showing the anomalous maps calculated from datasets collected at the Fe K-edge and below the Fe K-edge (left panel), and the anomalous maps calculated from datasets collected at the Zn K-edge and below the Zn K-edge (right panel). Anomalous maps calculated at K-edge and below K-edge are contoured at 5 σ and coloured in magenta and blue, respectively. All anomalous maps were calculated using a resolution range of 3.5-30 Å, from a set of datasets collected on the same crystal. Iron and zinc ions are shown as spheres coloured in red and grey, respectively.

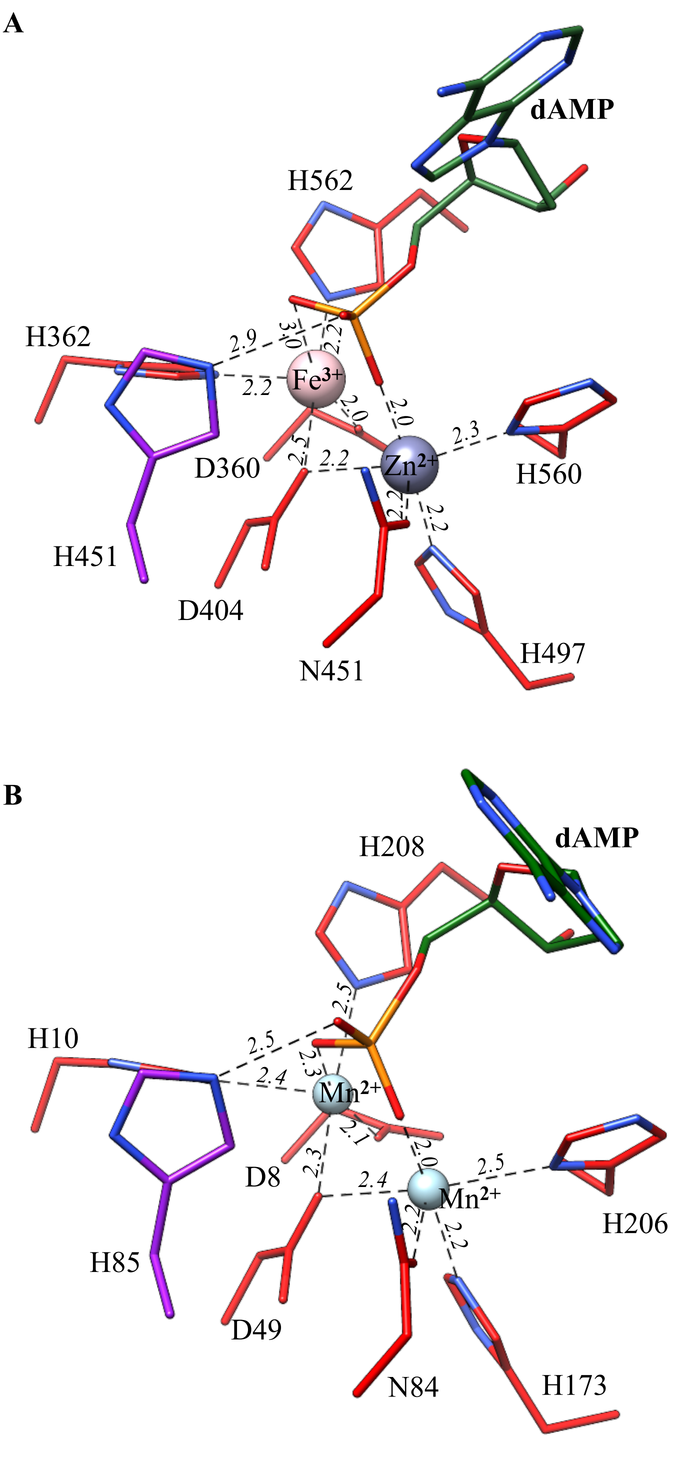

Supplementary Figure 4. Distances between metal ions, dAMP and protein residues in DP1 and Mre11 PDE domains. (A) Catalytic residues are shown as sticks, red for the metal-coordinating residues, purple for the proton-donnor. dAMP is shown in green. Catalytic metals are shown as spheres. Distances are measured in Å. (B) Same view for *P. furiosus* Mre11.

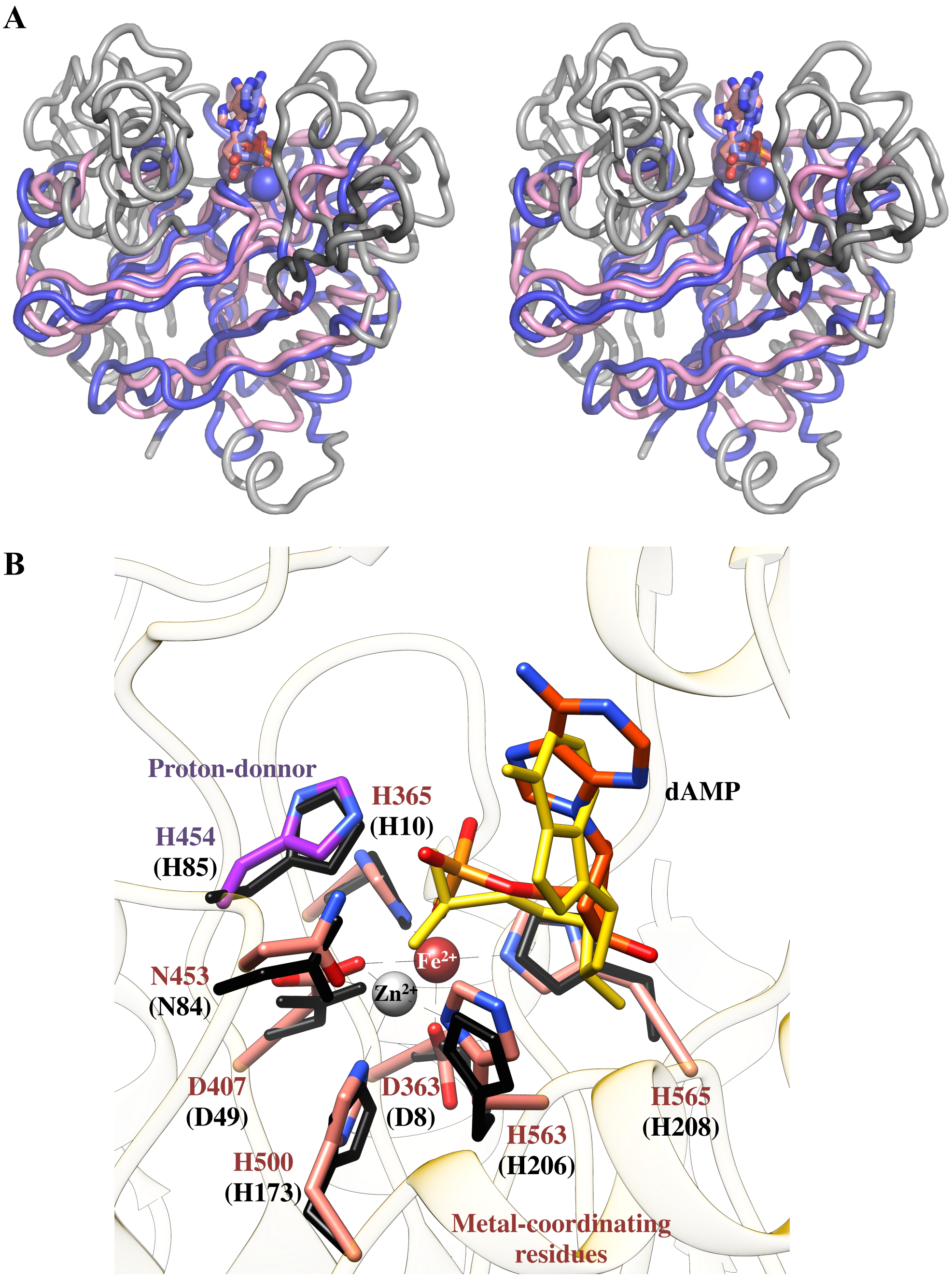

Supplementary Figure 5. Comparison of the DP1 and Mre11 PDE domains. (A) Cross-eye stereo-diagram of the N-terminal domain of the DP1 PDE domain superimposed on the PDE domain of *P. furiosus* Mre11. DP1 is drawn as a thin tube in blue, Mre11 in pink. Dissimilar regions are coloured in grey. Catalytic metals are shown as van der Waals spheres and dAMP as sticks. (B) Comparison of the DP1 and Mre11 nuclease active sites. Catalytic residues and dAMP are shown as sticks. For comparison, the corresponding residues and dAMP-bound molecule of the *P. furiosus* Mre11[3] active site superimposed on DP1 are shown in black and yellow, respectively.

Z score	Entry	rmsd	lali	nres	%id	Organism	Description
33,1	3e0j-E	3,0	353	408	19	*H. sapiens*	**DNAP δ B-subunit**
25,4	4y97-G	3,5	354	436	15	*H. sapiens*	**DNAP α B-subunit**
24,0	3flo-A	3,5	342	433	16	*S. cerevisiae*	**DNAP α B-subunit**
14,8	1s3m-A	14,8	161	165	20	*M. jananaschii*	Phosphodiesterase MJ0936
14,3	1wyd-A	14,3	117	428	16	*S. tokodaii*	Aspartyl-tRNA synthetase
13,4	1low-A	13,4	120	580	19	*T. thermophilus*	Aspartyl-tRNA synthetase
13,4	4wj3-M	10,4	117	589	19	*P. aeruginosa*	Glutamyl-tRNA emidotransferase
13,4	1eqr-A	11,5	116	590	17	*E. coli*	Aspartyl-tRNA synthetase
13,3	2xtiB	13,7	118	429	17	*B. malayi*	Asparaginyl-tRNA synthetase
13,1	3tho-B	3,2	177	366	16	*T. maritima*	Mre11
13,0	3av0-A	3,0	182	369	14	*M. jananaschii*	Mre11
13,0	2a22-A	2,8	170	193	18	*C. parvum*	Vacuolar Protein Sorting 29
13,0	4rmf-A	11,6	119	579	13	*M. smegmatis*	Aspartyl-tRNA synthetase
13,0	4tug-A	3,1	186	317	13	*M. jananaschii*	Mre11
13,0	1il2-B	11,4	117	585	16	*S. cerevisiae*	Aspartyl-tRNA synthetase
12,9	1ii7-A	3,1	183	333	18	*P. furiosus*	Mre11
12,9	3dsd-B	3,2	184	333	13	*P. furiosus*	Mre11
12,8	3t1i-C	3,1	192	377	14	*H. sapiens*	Mre11
12,8	4fbw-A	3,0	190	379	17	*S. pombe*	Mre11
12,8	4hd0-A	3,1	185	332	18	*P. furiosus*	Mre11

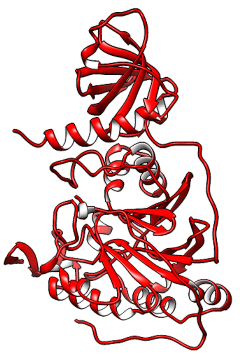

Dali search of the DP1 structure

Z score	Entry	rmsd	lali	nres	%id	Organism	Description
14,6	1wyd-A	1,6	85	428	20	*S. tokodaii*	Aspartyl-tRNA synthetase
13,6	2xti-B	1,4	82	429	23	*B. malayi*	Aspartyl-tRNA synthetase
13,4	1l0w-A	1,8	85	580	25	*T. thermophilus*	Aspartyl-tRNA synthetase
13,3	4wj3-M	1,7	85	589	24	*P. aeruginosa*	Glutamyl-tRNA synthetase
13,2	1eqr-A	1,8	85	590	21	*E. coli*	Aspartyl-tRNA synthetase
13,0	4wj4-A	1,7	85	586	24	*P. aeruginosa*	Aspartyl-tRNA synthetase
12,9	1x54-A	1,6	84	435	18	*P. horikoshii*	Aspartyl-tRNA synthetase
12,9	4o2d-A	1,7	83	581	18	*M. smegmatis*	Aspartyl-tRNA synthetase
12,7	1il2-A	1,9	85	585	21	*S. cerevisiae*	Aspartyl-tRNA synthetase
12,6	3nem-A	1,8	85	439	20	*T. kodakarensis*	Aspartyl-tRNA synthetase
12,4	1efw-A	1,9	85	580	25	*E. coli*	Aspartyl-tRNA synthetase
12,2	1e24-A	1,9	84	486	17	*E. coli*	Lysyl-tRNA synthetase
12,1	3e9h-D	1,8	84	485	17	*G. stearothermophilus*	Lysyl-tRNA synthetase
12,0	2z6k-B	1,8	77	126	22	*H. sapiens*	Replication protein A
11,9	1upa-A	2,1	85	515	18	*E. histolytica*	Lysyl-tRNA synthetase
11,9	3e0e-A	1,7	78	91	14	*M. maripaludis*	Replication protein A
11,7	1quq-A	1,8	77	121	22	*H. sapiens*	Replication protein A
11,7	3dm3-B	1,8	78	98	24	*M. jannaschii*	Replication protein A
9,9	1z9f-A	1,6	72	89	14	*T. maritima*	SSB protein
9,9	2dud-B	1,8	73	92	14	*H. sapiens*	Mitochondrial SSB protein
...	...	...	...	...	...	...	...
7,8	3e0j-A	2,2	70	416	17	*H. sapiens*	**DNAP δ B-subunit**
...	...	...	...	...	...	...	...
4,9	4y97-A	2,0	70	442	21	*H. sapiens*	**DNAP α B-subunit**

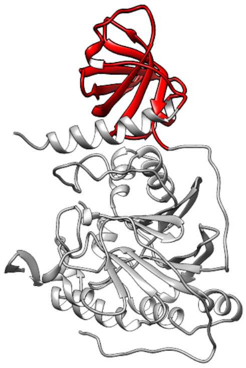

Dali search of the DP1 OB domain

Z score	Entry	rmsd	lali	nres	%id	Organism	Description
25,8	3e0j-A	2,5	264	416	19	*H. sapiens*	**DNAP δ B-subunit**
20,8	4y97-G	3,2	266	436	14	*H. sapiens*	**DNAP α B-subunit**
19,4	3flo-A	3,0	252	433	16	*S. cerevisiae*	**DNAP α B-subunit**
14,8	1s3m-B	2,5	161	165	20	*M. jananaschii*	Phosphodiesterase MJ0936
13,1	3tho-B	3,2	179	366	16	*T. maritima*	Mre11
13,0	2a22-A	2,8	170	193	18	*C. parvum*	Vacuolar Protein Sorting 29
13,0	3av0-A	3,0	182	369	14	*M. jananaschii*	Mre11
12,9	4tug-A	3,1	186	317	13	*M. jananaschii*	Mre11
12,9	1ii7-A	3,1	183	333	18	*P. furiosus*	Mre11
12,9	3dsd-B	3,1	183	333	13	*P. furiosus*	Mre11
12,7	3t1i-C	3,1	191	377	14	*H. sapiens*	Mre11
12,7	4fbw-A	3,0	190	379	17	*S. pombe*	Mre11
12,6	4fbq-A	3,1	193	418	17	*S. pombe*	Nbs1-Mre11 complex
12,8	4hd0-A	3,1	185	332	18	*P. furiosus*	Mre11
12,6	1s8e-B	3,2	184	333	17	*P. furiosus*	Mre11-3
12,5	3lh7-A	3,1	183	333	18	*M. musculus*	Vacuolar Protein Sorting 29
12,3	1uf3-F	3,0	167	228	12	*T. thermophilus*	TT1561
12,2	2zo9-C	3,1	177	271	15	*E. aerogenes*	glycerophosphodiesterase
12,1	3ib7-A	3,6	178	296	17	*M. tuberculosis*	Rv0805
12,1	4yke-A	3,0	191	404	16	*C. thermophilum*	Mre11

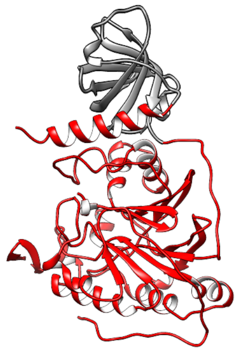

Dali search of the DP1 PDE domain

Supplementary Figure 6. Dali[4] search of the DP1 structure against the Protein Data Bank. Top twenty hits of DALI of the DP1 structure (top), DP1-OB domain (middle) and DP1-PDE domain (bottom) against the Protein Data Bank. Chain A was used in the search but comparable results were obtained with chain B. Eukaryotic DNA polymerases B-subunits are coloured in blue.

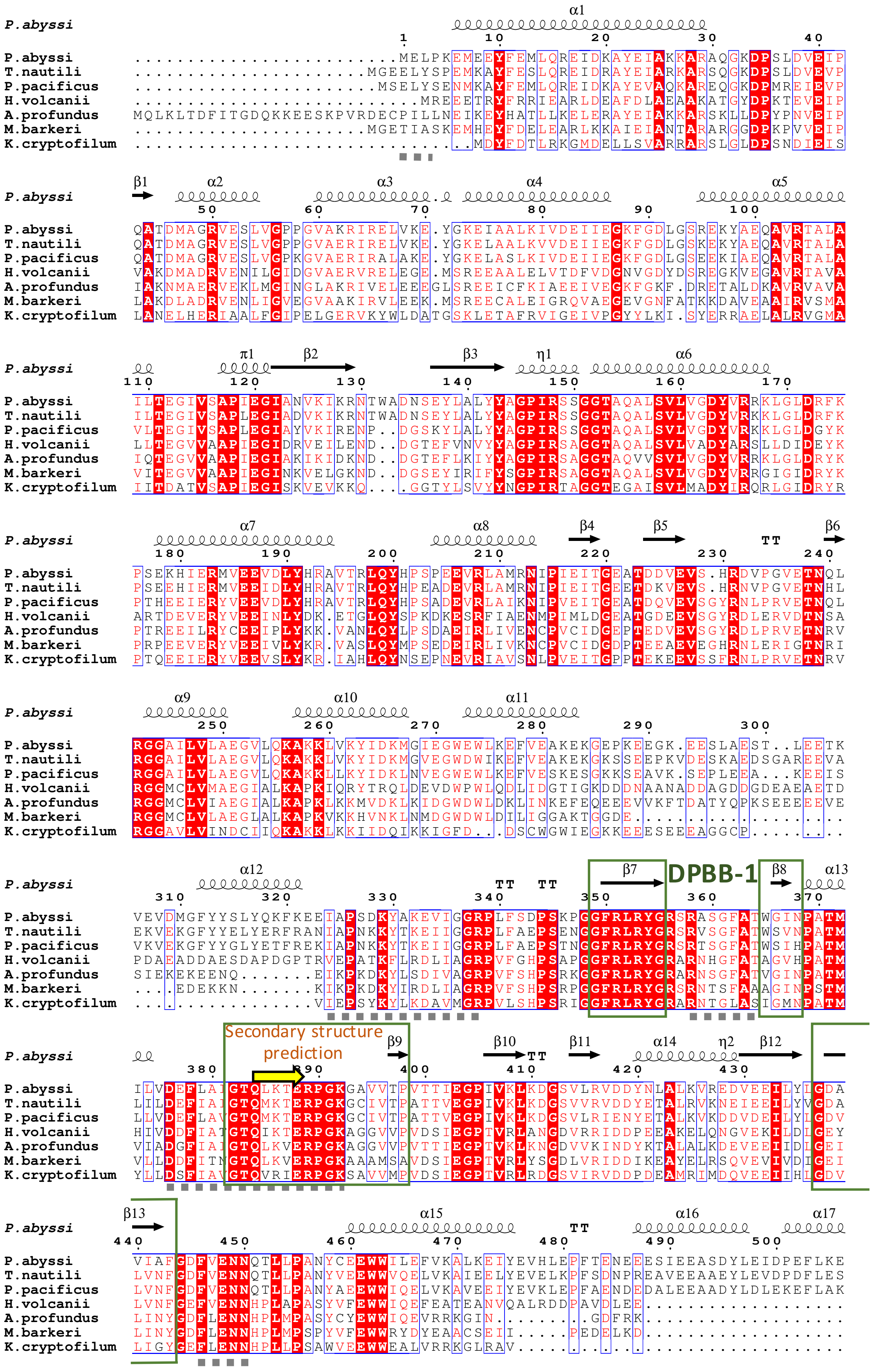
P.abyssi
T.nautili
P.pacificus
H.volcanii
A.profundus
M.barkeri
K.cryptofilum
DPBB-1
Secondary structure prediction

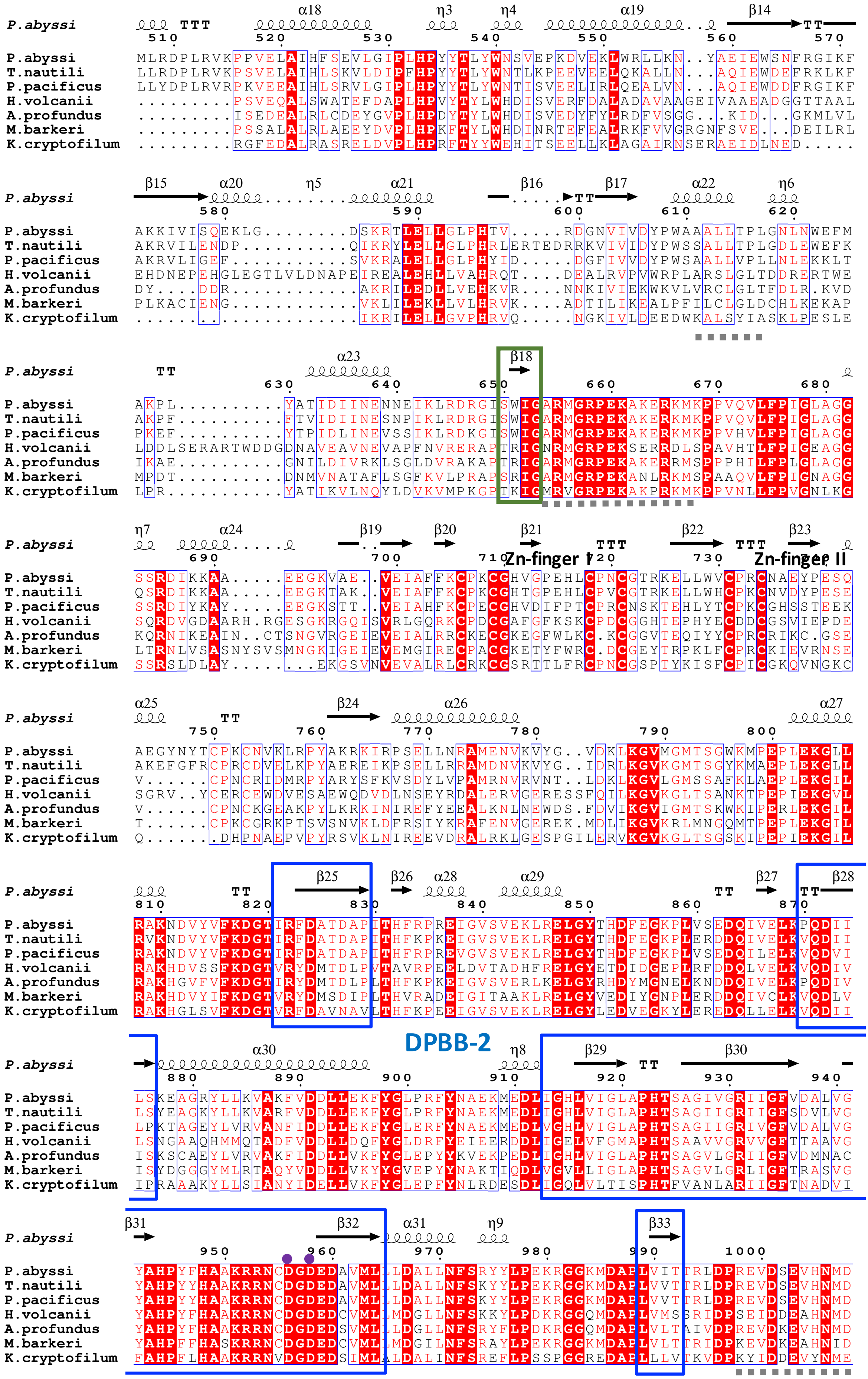

P.abyssi
T.nautili
P.pacificus
H.volcanii
A.profundus
M.barkeri
K.cryptofilum
Zn-finger I
Zn-finger II
DPBB-2

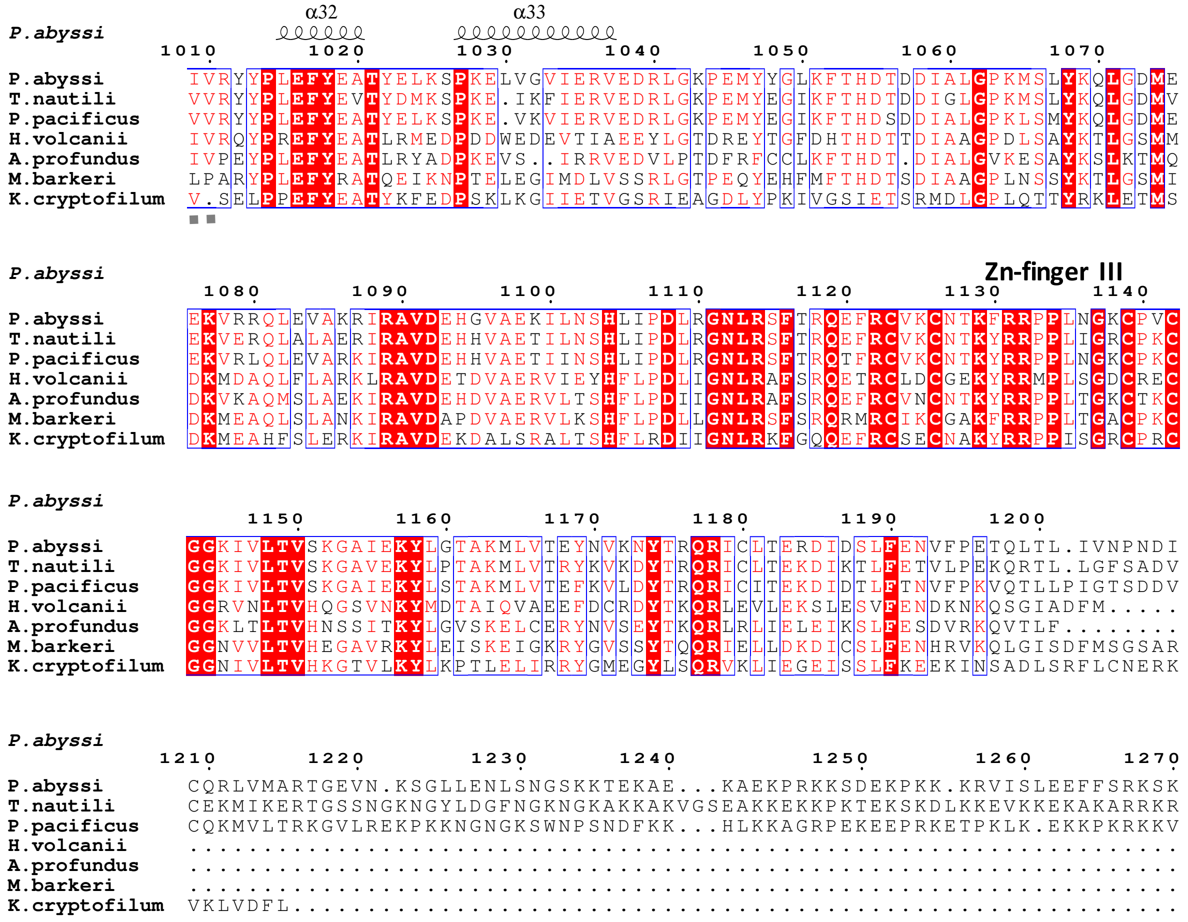

Supplementary Figure 7: Alignment of DP2 structure amino acid sequence with other DP2 subunits. MultAlin[1] and ESPript[2] 3.0 were used to generate an alignment with the following proteins (GI accession number): *Pyrococcus abyssi* (504648395), *Thermococcus nautili* (757137858), *Paleococcus pacificus* (851300592), *Haloferax volcanii* (490144762), *Archaeoglobus profondus* (502705717), *Methanosarcina barkeri* (851209399), *Korarchaeum cryptofilum* (501267152). Conserved residues are boxed in red and highly conserved residues are coloured in red. Secondary structure elements observed in *P. abyssi* DP2 structure are also shown. The catalytic double-psi beta-barrel subdomains DPBB-1 and DPBB-2 are highlighted with green and blue boxes, respectively. Dashed lines indicate regions of the protein, which were not seen in the electron density and not included in the final model. A yellow arrow shows the result of a secondary structure prediction[5], which suggests the presence of a β-strand in a disordered region of the protein; this β-strand might be part of the DPBB-1 subdomain. Catalytic aspartic residues are outlined by purple circles.

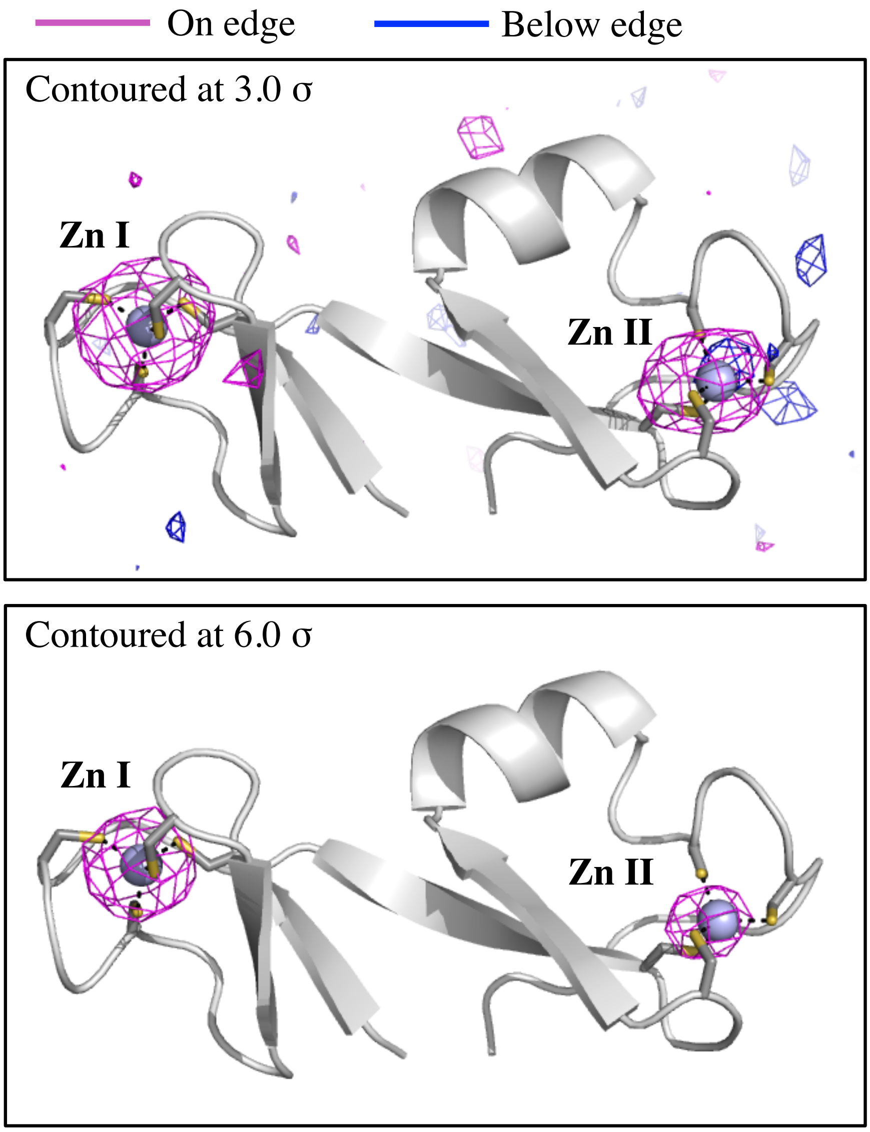

Supplementary Figure 8: DP2 contains two zinc metal-ion binding motifs. Validation of metal-binding sites by comparing anomalous maps calculated from datasets collected at different energies. Close-view of zinc metal-ion binding motifs I and II. The anomalous maps calculated from datasets collected at the Zn K-edge (9671 eV – 1.2820 Å) and below the Zn K-edge (9625 eV – 1.2882 Å) are shown as mesh in magenta and blue, respectively. Anomalous maps are calculated using a resolution range of 3.5-30 Å and contoured at 3 σ (top panel) or 6 σ (bottom panel). DP2 is shown in ribbon, the cysteine ligands as sticks and zinc ions as spheres.

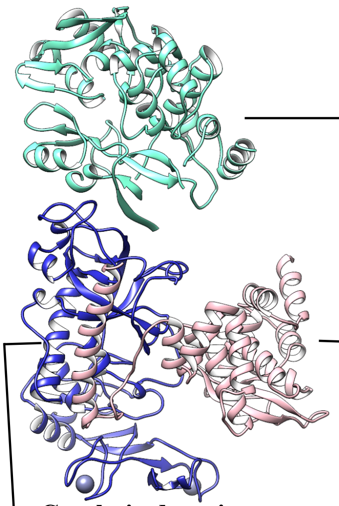

Central domain: No hit with Z-score > 4,0

NTD

Z score	Entry	rmsd	lali	nres	%id	Organism	Description
42,7	3o59-X	0,5	241	244	94	*P. horikoshii*	PolD NTD-DP2

Catalytic domain

Z score	Entry	rmsd	lali	nres	%id	Organism	Description
6,0	4mex-J	5,1	126	1143	15	*E. coli*	RNAP β' subunit
5,9	5flm-A	5,1	135	1427	11	*B. taurus*	RNAP II RPB1 subunit
5,9	4xsx-D	5,3	138	1166	14	*E. coli*	RNAP β' subunit
5,8	4bbs-A	5,9	140	1419	10	*S. cerevisiae*	RNAP II RPB1 subunit
5,8	4ylp-D	6,3	146	1362	13	*E. coli*	RNAP β' subunit
5,7	3hox-A	4,7	134	1417	7	*S. cerevisiae*	RNAP II RPB1 subunit
5,7	4yln-J	5,2	134	1362	14	*E. coli*	RNAP β' subunit
5,7	4v1n-A	5,5	139	1422	9	*S. cerevisiae*	RNAP II RPB1 subunit
5,7	4ylp-J	6,3	148	1362	14	*E. coli*	RNAP β' subunit
5,6	4c3h-A	5,4	136	1523	10	*S. cerevisiae*	RNAP I A190 subunit
5,6	1k83-A	4,6	124	1366	8	*S. cerevisiae*	RNAP II RPB1 subunit
5,6	4yfk-D	4,9	135	1163	14	*E. coli*	RNAP β' subunit
5,6	3m4o-A	4,9	134	1395	7	*S. cerevisiae*	RNAP II RPB1 subunit
5,6	4ylo-D	5,2	134	1362	14	*E. coli*	RNAP β' subunit
5,6	2nvq-A	5,3	136	1405	10	*S. cerevisiae*	RNAP II RPB1 subunit
5,6	3hkz-I	5,5	133	836	8	*S. solfataricus*	RNAP RPOL subunit
5,6	4a3g-A	5,9	141	1422	9	*S. cerevisiae*	RNAP II RPB1 subunit
5,5	4c2m-A	6,5	146	1521	10	*S. cerevisiae*	RNAP I A190 subunit
5,5	3s1n-A	4,9	133	1405	7	*S. cerevisiae*	RNAP II RPB1 subunit
5,5	4yfn-D	5,1	130	1163	15	*E. coli*	RNAP β' subunit
5,5	2yu9-A	5,3	136	1411	10	*S. cerevisiae*	RNAP II RPB1 subunit
5,5	2a69-D	5,3	142	1392	13	*T. thermophilus*	RNAP β' subunit
5,4	4c3i-A	5,8	142	1484	10	*S. cerevisiae*	RNAP I A190 subunit
5,4	4qiw-A	4,2	116	863	8	*T. kodakarensis*	RNAP RPOL subunit
5,4	1twf-A	4,7	135	1419	10	*S. cerevisiae*	RNAP II RPB1 subunit
5,4	2pmz-Q	5,3	133	776	9	*S. solfataricus*	RNAP RPOL subunit
5,4	4fer-B	3,2	94	207	6	*B. subtilis*	Expansin
5,4	1r9s-A	4,9	135	1381	7	*S. cerevisiae*	RNAP II RPB1 subunit
5,4	4b1p-W	5,5	131	872	10	*S. shibatae*	RNAP RPOL subunit
5,4	4s20-I	5,3	134	1160	13	*E. coli*	RNAP β' subunit
5,4	2b63-A	5,5	137	1416	10	*S. cerevisiae*	RNAP II RPB1 subunit
5,4	2pmz-A	5,3	132	776	8	*S. solfataricus*	RNAP RPOL subunit
5,4	2cw0-D	5,3	140	1392	13	*T. thermophilus*	RNAP β' subunit
5,4	4by7-A	6,0	140	1426	10	*S. cerevisiae*	RNAP II RPB1 subunit
5,4	4gzz-D	5,8	142	1358	13	*T. thermophilus*	RNAP β' subunit
5,4	4x67-A	5,6	138	1394	9	*S. cerevisiae*	RNAP II RPB1 subunit
5,4	2o5i-N	5,0	139	1303	14	*T. thermophilus*	RNAP β' subunit
5,4	3qc8-A	3,2	87	170	10	*H. sapiens*	Trans. End. Ret. ATPase
5,4	4q4z-D	6,1	145	1494	13	*T. thermophilus*	RNAP β' subunit
5,3	1r9t-A	4,9	134	1395	7	*S. cerevisiae*	RNAP II RPB1 subunit
5,3	3d30-A	3,1	94	208	6	*B. subtilis*	Expansin-like
5,3	4xsy-D	4,9	136	1166	14	*E. coli*	RNAP β' subunit
5,3	3j0k-A	4,8	135	1426	7	*H. sapiens*	RNAP II RPB1 subunit
...	...	...	...	...	...	...	...
5,3	1ynj-D	5,1	132	1238	14	*T. aquaticus*	RNAP β' subunit
...	...	...	...	...	...	...	...
5,0	1n10-A	3,8	103	228	10	*P. pratense*	Pollen allergen PHL P1
...	...	...	...	...	...	...	...
4,0	2j7o-A	3,7	108	934	10	*N. crassa*	RNAP QDE1-like
...	...	...	...	...	...	...	...

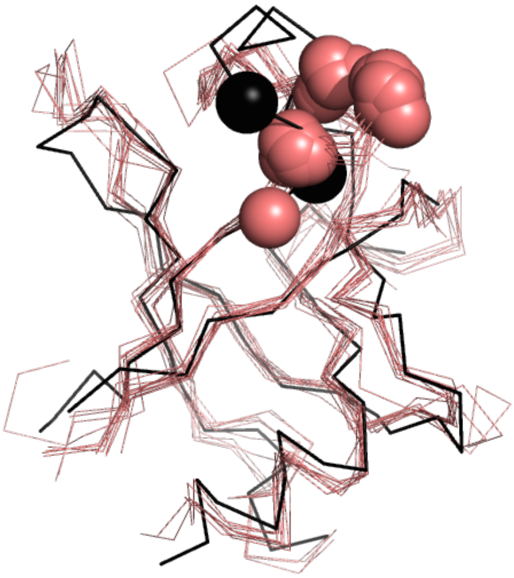

Supplementary Figure 9. Dali[4] search of the NTD-, catalytic- and central- domains of DP2 structure against the Protein Data Bank. The PolD double-psi beta-barrel (DPBB-2) (black) is superimposed onto the double-psi beta-barrels (DPBB-A) of 11 representative RNA polymerases structures identified from the Dali search (red): RNAP β’ subunit from *E. coli* (4MEX[6]), RNAP II *RPB1* subunit from *B. taurus* (5FLM[7]), RNAP II *RPB1* subunit from *S. cerevisiae* (4BBS[8]), RNAP I *A190* subunit from *S. cerevisiae* (4C3H[9]), RNAP *RPOL* subunit

from *S. solfataricus* (3HKZ[10]), RNAP β' subunit from *T. thermophilus* (2A69[11]), RNAP *RPOL* subunit from *T. kodakarensis* (4QIW[12]), RNAP *RPOL* subunit from *S. shibatae* (4BLP[13]), RNAP II *RPB1* subunit from *H. sapiens* (3J0K[7]), RNAP β' subunit from *T. aquaticus* (1YNJ[14]) and RNAP QDE1-like from *N. crassa* (2J7O[15]). Cα of the catalytic aspartic residues are shown as spheres (red for RNAPs, black for PolD).

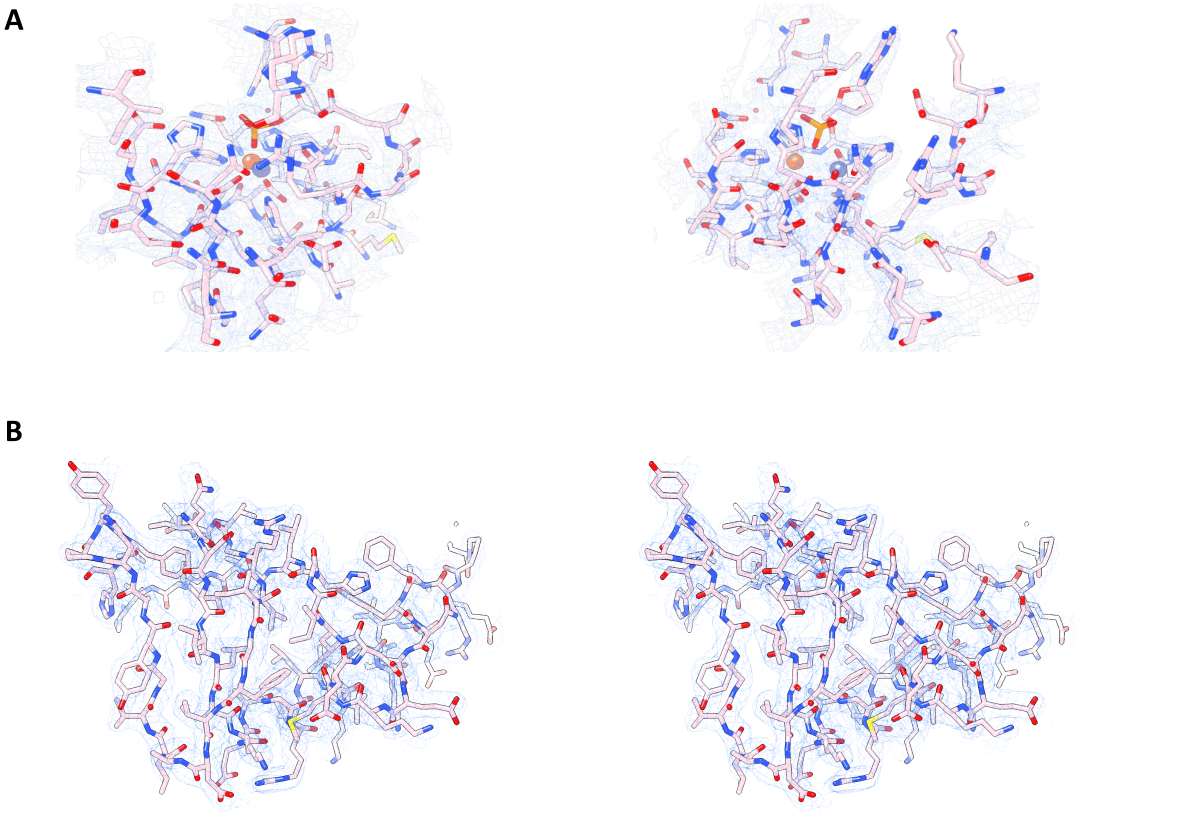

Supplementary Figure 10. Illustration of the quality of the electron density. Stereo images of a portion of the 2Fo-Fc electron density map contoured at 1.0 σ for both DP1 (A) and DP2 (B) crystal structures.

References

1. Corpet, F., Gouzy, J. & Kahn, D. Browsing protein families via the 'Rich Family Description' format. *Bioinforma. Oxf. Engl.* **15,** 1020–1027 (1999).
2. Robert, X. & Gouet, P. Deciphering key features in protein structures with the new ENDscript server. *Nucleic Acids Res.* **42,** W320–W324 (2014).
3. Hopfner, K. P. *et al.* Structural biochemistry and interaction architecture of the DNA double-strand break repair Mre11 nuclease and Rad50-ATPase. *Cell* **105,** 473–485 (2001).
4. Holm, L. & Rosenström, P. Dali server: conservation mapping in 3D. *Nucleic Acids Res.* **38,** W545–W549 (2010).
5. Kelley, L. A., Mezulis, S., Yates, C. M., Wass, M. N. & Sternberg, M. J. E. The Phyre2 web portal for protein modeling, prediction and analysis. *Nat. Protoc.* **10,** 845–858 (2015).
6. Degen, D. *et al.* Transcription inhibition by the depsipeptide antibiotic salinamide A. *eLife* **3,** e02451 (2014).
7. Bernecky, C., Herzog, F., Baumeister, W., Plitzko, J. M. & Cramer, P. Structure of transcribing mammalian RNA polymerase II. *Nature* **529,** 551–554 (2016).
8. Sainsbury, S., Niesser, J. & Cramer, P. Structure and function of the initially transcribing RNA polymerase II-TFIIB complex. *Nature* **493,** 437–440 (2013).
9. Fernández-Tornero, C. *et al.* Crystal structure of the 14-subunit RNA polymerase I. *Nature* **502,** 644–649 (2013).
10. Hirata, A., Klein, B. J. & Murakami, K. S. The X-ray crystal structure of RNA polymerase from Archaea. *Nature* **451,** 851–854 (2008).
11. Artsimovitch, I. *et al.* Allosteric modulation of the RNA polymerase catalytic reaction is an essential component of transcription control by rifamycins. *Cell* **122,** 351–363 (2005).
12. Jun, S.-H. *et al.* The X-ray crystal structure of the euryarchaeal RNA polymerase in an open-clamp configuration. *Nat. Commun.* **5,** 5132 (2014).
13. Wojtas, M. N., Mogni, M., Millet, O., Bell, S. D. & Abrescia, N. G. A. Structural and functional analyses of the interaction of archaeal RNA polymerase with DNA. *Nucleic Acids Res.* **40,** 9941–9952 (2012).
14. Campbell, E. A. *et al.* Structural, functional, and genetic analysis of sorangicin inhibition of bacterial RNA polymerase. *EMBO J.* **24,** 674–682 (2005).
15. Salgado, P. S. *et al.* The structure of an RNAi polymerase links RNA silencing and transcription. *PLoS Biol.* **4,** e434 (2006).
